# A Unified Information Bottleneck Framework for Multimodal Biomedical Machine Learning

**DOI:** 10.3390/e28040445

**Published:** 2026-04-14

**Authors:** Liang Dong

**Affiliations:** 1 Department of Electrical and Computer Engineering, Baylor University, Waco, TX 76798, USA; liang_dong@baylor.edu; 2 Department of Radiology, The University of Texas Southwestern Medical Center, Dallas, TX 75390, USA; liang.dong@utsouthwestern.edu

**Keywords:** information bottleneck, mutual information, entropy, multimodal learning, biomedical machine learning, multi-omics, transfer entropy, missing modalities, representation learning, uncertainty quantification, foundation models

## Abstract

Multimodal biomedical machine learning increasingly integrates heterogeneous data sources (including medical imaging, multi-omics profiles, electronic health records, and wearable sensor signals) to support clinical diagnosis, prognosis, and treatment response prediction. Despite strong empirical performance, most existing multimodal systems lack a principled theoretical foundation for understanding why fusion improves prediction, how information is distributed across modalities, and when models can be trusted under incomplete or shifting data. This paper develops a unified information-theoretic framework that formalizes multimodal biomedical learning as an information optimization problem. We formulate multimodal representation learning through the information bottleneck principle, deriving a variational objective that balances predictive sufficiency against informational compression in an architecture-agnostic manner. Building on this foundation, we introduce information-theoretic tools for decomposing modality contributions via conditional mutual information, quantifying redundancy and synergy, and diagnosing fusion collapse. We further show that robustness to missing modalities can be cast as an information consistency problem and extend the framework to longitudinal disease modeling through transfer entropy and sequential information bottleneck objectives. Applications to multimodal foundation models, uncertainty quantification, calibration, and out-of-distribution detection are developed. Empirical case studies across three biomedical datasets (TCGA breast cancer multi-omics, TCGA glioma clinical-plus-molecular data, and OASIS-2 longitudinal Alzheimer’s data) show that the framework’s key quantities are computable and interpretable on real data: MI decomposition identifies modality dominance and redundancy; the VMIB traces a compression–prediction tradeoff in the information plane; entropy-based selective prediction raises accuracy from 0.787 to 0.939 at 50% coverage; transfer entropy reveals stage-dependent modality influence in disease progression; and pretraining/adaptation diagnostics distinguish efficient from wasteful fine-tuning strategies. Together, these results develop entropy and mutual information as organizing principles for the design, analysis, and evaluation of multimodal biomedical AI systems.

## 1. Introduction

The biomedical data ecosystem has entered an intrinsically multimodal era. Contemporary biomedical discovery and clinical decision-making increasingly rely on the joint use of heterogeneous data sources, including medical imaging and digital pathology, multi-omics profiles, electronic health records (EHR), clinical narratives, laboratory tests, and continuous signals from wearable and ambient sensors [1,2,3]. For many tasks, including disease diagnosis, prognosis, treatment response prediction, and population-level risk stratification, no single modality provides a complete or reliable view of the underlying biological state [4,5]. As a result, artificial intelligence (AI) and machine learning (ML) systems in biomedicine are now expected to integrate diverse modalities to extract complementary information and support robust, clinically meaningful inference [6,7].

Recent advances in deep neural networks, attention-based fusion mechanisms, graph neural networks, and large multimodal foundation models have demonstrated strong empirical performance across a range of biomedical tasks, from cancer outcome prediction to longitudinal disease modeling [7,8,9]. However, most multimodal biomedical AI systems remain largely empirically engineered [10]. Typical pipelines are designed by selecting modality-specific encoders, applying heuristic fusion strategies, and optimizing end-to-end predictive accuracy. While effective in controlled settings, such approaches often provide limited insight into why multimodal integration works, which modalities truly contribute predictive value, and how model behavior changes under distribution shift, missing data, or heterogeneous clinical environments [11,12].

These limitations are particularly consequential in biomedical and clinical contexts, where AI systems must operate under stringent requirements for robustness, interpretability, and trustworthiness [13,14]. Data are frequently incomplete or unevenly sampled; modalities may be missing, delayed, or corrupted; and patient populations may differ substantially across institutions, devices, and time periods. Moreover, clinical decision-making requires not only accurate predictions but also principled uncertainty estimates and transparent reasoning about model confidence [15,16]. Empirical multimodal architectures alone provide little guidance on how to address these challenges in a systematic and theoretically grounded manner.

Entropy and information theory offer a natural and powerful lens through which to address these issues. Information-theoretic concepts provide a principled language for characterizing what is learned from data, what is discarded, and how predictive information is distributed across modalities and representations [17,18]. Historically, entropy-based methods have played an important role in biomedical engineering and data analysis, particularly in signal complexity analysis and feature extraction [19,20]. The information bottleneck (IB) principle, introduced by Tishby et al. [21], has been applied to representation learning in deep networks [22] and extended to variational settings [23], while partial information decomposition has formalized notions of redundancy and synergy among random variables [24]. More recently, contrastive learning objectives, including InfoNCE [25], SimCLR [26], and CLIP [27], have been shown to maximize lower bounds on mutual information between views or modalities, establishing a direct link between self-supervised representation learning and information optimization. Neural mutual information estimators [28,29] have made it feasible to evaluate information-theoretic quantities in high-dimensional spaces, while IB variants including the deterministic IB [30] and conditional entropy bottleneck [31] have expanded the theoretical toolkit available for representation analysis [32,33,34].

Surveys on multimodal machine learning [10,35] and multimodal fusion strategies [3,36,37] have catalogued architectural approaches, including attention-based mechanisms [38,39] and transformer-based fusion [37]. In the biomedical domain, multimodal integration has achieved notable results in precision oncology [40,41], computational pathology [7,42], multimodal graph learning [43], and longitudinal disease modeling [44]. Recent work has explored information-theoretic views of deep learning [22,45] and the multi-view IB for robust representations [46]. However, these lines of research have remained largely separate. Existing IB surveys focus primarily on unimodal settings [32] and do not address the specific challenges of multimodal biomedical data, including heterogeneous modality structures, systematic missing data, longitudinal disease dynamics, and the unique trust requirements of clinical deployment. Similarly, multimodal learning reviews emphasize architectural choices rather than foundational principles that govern when and why fusion succeeds.

This paper advances the central thesis that multimodal biomedical machine learning is fundamentally an information optimization problem. From this perspective, learning can be viewed as the construction of latent representations that balance two competing objectives: preserving information that is predictive of clinically relevant targets, while compressing away nuisance variability, noise, and confounding factors inherent in high-dimensional biomedical data. Multimodality introduces additional structure, raising fundamental questions about how information from different sources should be combined, how redundancy and synergy between modalities should be quantified, and how predictive performance degrades when modalities are partially observed or absent.

**Figure 1:**
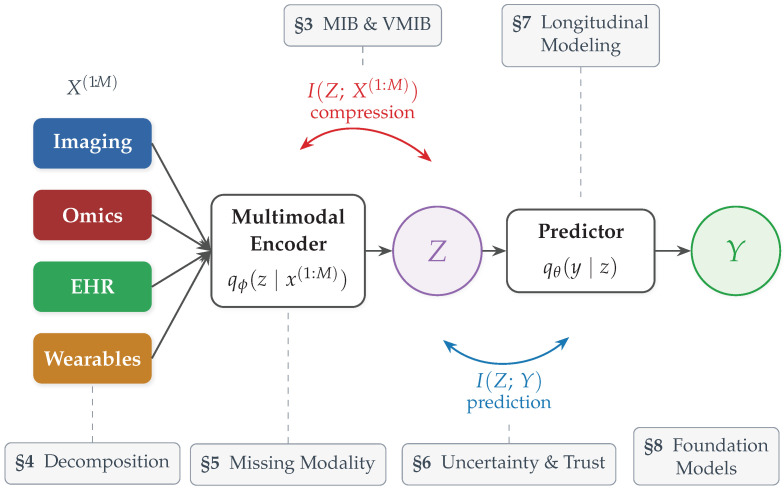
Unified information-theoretic framework for multimodal biomedical machine learning. Heterogeneous biomedical modalities $X^{\left(1:M\right)}$ (imaging, omics, electronic health records, and wearable signals) are mapped through a multimodal encoder $q_{\phi }\left(z|x^{\left(1:M\right)}\right)$ to a compressed latent representation *Z*, which feeds a predictor $q_{\theta }\left(y|z\right)$ for clinical target *Y*. The MIB objective balances the competing goals of compression $I(Z;\;X^{\left(1:M\right)})$ (red arc) and prediction I(Z;Y) (blue arc). Section badges indicate where each theoretical component of the framework is developed: formulation and variational optimization (§3), information decomposition (§4), missing-modality robustness (§5), uncertainty and calibration (§6), longitudinal modeling (§7), and foundation model diagnostics (§8). Values and curves are illustrative.

The specific contributions of this paper are as follows. First, we present a unified formulation of multimodal biomedical learning through the multimodal information bottleneck (MIB) objective and its tractable variational approximation (the VMIB), showing how representation learning, regularization, and modality fusion can be understood under a single information-theoretic principle, and how existing multimodal architectures, from early and late fusion to attention-based and graph-based models, map onto this framework (Section 3). Second, we survey the information decomposition landscape, reviewing partial information decomposition and introducing operationally grounded proxies for redundancy and synergy together with a diagnostic for fusion collapse that connects information-theoretic quantities to the learned representation space (Section 4). Third, we review missing-modality robustness through the lens of information consistency, examining how KL-based training objectives provide a principled alternative to ad hoc imputation and modality dropout heuristics (Section 5). Fourth, we cover uncertainty quantification, calibration, and out-of-distribution detection from an entropy-based perspective, including connections to conformal prediction for distribution-free guarantees (Section 6). Fifth, we extend the framework to longitudinal disease modeling via a sequential information bottleneck coupled with transfer entropy, enabling temporal modality prioritization (Section 7). Sixth, we provide information-theoretic diagnostics for multimodal foundation models, including representation entropy, modality retention, and information-efficient adaptation criteria, that are architecture-agnostic and applicable to emerging large-scale biomedical AI systems (Section 8). Seventh, we present comprehensive empirical validation across three biomedical datasets (TCGA breast cancer multi-omics, TCGA glioma clinical-plus-molecular data, and OASIS-2 longitudinal Alzheimer’s data), illustrating that the framework’s key quantities are computable, interpretable, and practically useful on real biomedical data (Section 9). Finally, we identify ten open research frontiers, including fairness, federated learning, and domain adaptation, and discuss how information theory provides a unifying language for these emerging challenges (Section 10). Throughout, we illustrate the framework on both a synthetic two-modality example (Section 3.8) and the real-data case study (Section 9). By synthesizing these perspectives, this paper develops entropy and information theory as organizing principles for the design, analysis, and evaluation of multimodal biomedical ML systems. Figure 1 provides an overview of the framework and its components.

## 2. Notation and Information-Theoretic Preliminaries

We briefly fix notation and recall the information-theoretic quantities used throughout. Readers familiar with these concepts may proceed to Section 3; a concise reference is provided in Table 1.

Let *X* be a random variable on a measurable space X with distribution p(x). The Shannon entropy $H\left(X\right)≜-E_{p(x)}\left[\log p\left(x\right)\right]$ quantifies the intrinsic uncertainty of *X*, while the conditional entropy $H\left(Y|X\right)≜-E_{p(x,y)}\left[\log p\left(y|x\right)\right]$ measures the residual uncertainty of *Y* after observing *X* [17,18]. In supervised learning, H(Y∣X) equals the Bayes-optimal expected log-loss and represents the irreducible uncertainty in predicting *Y* from *X*; it lower-bounds the expected log-loss of any predictor, and, via Fano’s inequality, also constrains classification error rates [18].

Mutual information (MI) measures the statistical dependence between *X* and *Y* and admits two equivalent forms

(1) $$I\left(X;Y\right)=H\left(Y\right)-H\left(Y|X\right)=\mathrm{KL}\left(p(x,y)\;∥\;p(x)p(y)\right),$$

where KL(·∥·) denotes the Kullback–Leibler divergence [18,47]. MI is nonnegative, symmetric, and invariant to invertible transformations, making it well suited for quantifying predictive relevance across heterogeneous biomedical modalities. The conditional mutual information $I\left(X^{\left(i\right)};Y|X^{\left(j\right)}\right)=H\left(Y|X^{\left(j\right)}\right)-H\left(Y|X^{\left(i\right)},X^{\left(j\right)}\right)$ measures the incremental predictive value of modality $X^{\left(i\right)}$ given $X^{\left(j\right)}$, and is central to understanding redundancy and complementarity across data sources [18].

The KL divergence belongs to the broader class of *f*-divergences $D_{f}\left(p∥q\right)=\int q\left(x\right)f\left(p\left(x\right)/q\left(x\right)\right)\;dx$, which includes the total variation distance, $\chi^{2}$-divergence, and Hellinger distance as special cases [48]. Similarly, the Rényi entropy $H_{\alpha }\left(X\right)={\left(1-\alpha \right)}^{-1}\log\sum_{x}p{\left(x\right)}^{\alpha }$ generalizes Shannon entropy (α→1), with special cases including the Hartley entropy (α=0), collision entropy (α=2), and min-entropy (α→∞) [49]. While this paper focuses primarily on Shannon-theoretic quantities, *f*-divergences appear naturally in variational bounds (Section 3.5) and in generative adversarial formulations [50], while Rényi entropy provides tighter characterizations of tail behavior relevant to out-of-distribution detection (Section 6.2).

A fundamental constraint on information processing is the data processing inequality: for any representation *Z* that is a (possibly stochastic) function of *X* alone, forming the Markov chain Y→X→Z, we have I(Z;Y)≤I(X;Y) [18]. This inequality has profound implications for representation learning: no deterministic or stochastic transformation of the data can create predictive information that was not present in the original observations. In the multimodal setting, the data processing inequality implies that the representation *Z* can contain at most as much information about *Y* as is jointly present in all modalities, establishing a fundamental ceiling on fusion performance. The geometry of these constraints is formalized through information geometry [51], which provides a Riemannian structure on the space of probability distributions and offers geometric interpretations of learning dynamics.

Estimating MI in high-dimensional continuous spaces poses significant practical challenges. Classical nonparametric estimators based on *k*-nearest neighbors [52] scale poorly to the dimensionality typical of biomedical data (e.g., high-resolution imaging, whole-genome sequencing). Neural MI estimators, including MINE [28] and its variants, use parametric critics to approximate MI via variational bounds, achieving scalability at the cost of estimation variance [29]. The choice of bound (e.g., Donsker–Varadhan, noise-contrastive, tractable variational) introduces bias–variance trade-offs that affect downstream use in IB objectives. These estimation challenges motivate the variational approach developed in Section 3.

The information bottleneck (IB) principle [21] seeks a representation *Z* of input *X* that solves

(2) $$\min_{p(z|x)}\;I\left(Z;X\right)-\beta \;I\left(Z;Y\right),$$

where β>0 controls the trade-off between compression (I(Z;X) small) and prediction (I(Z;Y) large). A representation *Z* is sufficient for predicting *Y* from *X* when I(Z;Y)=I(X;Y), and minimal sufficient when it additionally minimizes I(Z;X) among all sufficient representations [18,21].

For temporal processes, the transfer entropy from $\left\{X_{t}\right\}$ to $\left\{Y_{t}\right\}$ is defined as

(3) $${\mathrm{TE}}_{X\to Y}≜I\left(X_{t-k:t-1};\;Y_{t}|Y_{t-\ell :t-1}\right),$$

where $X_{t-k:t-1}=\left(X_{t-k},\ldots ,X_{t-1}\right)$ and $Y_{t-\ell :t-1}=\left(Y_{t-\ell },\ldots ,Y_{t-1}\right)$ denote finite histories of length *k* and *ℓ*, respectively [53]. Transfer entropy captures the additional predictive information that the past of *X* provides about the current state of *Y* beyond *Y*’s own history, and unlike symmetric measures such as MI, it quantifies directional and potentially nonlinear temporal dependencies [53,54].

## 3. The Multimodal Information Bottleneck: Formulation and Variational Optimization

We now formalize multimodal biomedical machine learning as an information optimization problem and derive a tractable variational objective, providing a unifying theoretical lens that connects representation learning, modality fusion, robustness, and generalization while remaining agnostic to specific model architectures.

### 3.1. Problem Setup

Let $\left\{X^{\left(1\right)},\ldots ,X^{\left(M\right)}\right\}$ denote *M* heterogeneous biomedical modalities, where each $X^{\left(i\right)}$ takes values in a space $X^{\left(i\right)}$, and let Y∈Y denote a target variable of interest. We assume data are generated according to an unknown joint distribution

(4) $$\left(X^{\left(1\right)},\ldots ,X^{\left(M\right)},Y\right)∼p^{⋆}\left(x^{\left(1\right)},\ldots ,x^{\left(M\right)},y\right).$$

A multimodal learning system constructs a latent representation Z∈Z via an encoder $p(z|x^{\left(1\right)},\ldots ,x^{\left(M\right)})$ and predicts *Y* through p(y∣z). The variable *Z* may represent a joint embedding, a disease state, or any abstract representation; we impose no parametric assumptions on these conditional distributions.

### 3.2. Multimodal Sufficiency and Minimality

The primary goal of representation learning is to preserve information relevant for predicting *Y*. This leads to two complementary desiderata.

**Definition 1**  (Multimodal Sufficiency)**.** *A representation Z is sufficient for predicting Y from ${\left\{X^{\left(i\right)}\right\}}_{i=1}^{M}$ if*

(5) $$I\left(Z;Y\right)=I\left(X^{\left(1\right)},\ldots ,X^{\left(M\right)};Y\right).$$

Sufficiency is a statement about the joint distribution of $\left(X^{\left(1:M\right)},Y,Z\right)$, not about any particular predictor. Because *Z* is constructed as a (possibly stochastic) function of $X^{\left(1:M\right)}$ alone, the Markov chain $Y\to X^{\left(1:M\right)}\to Z$ holds: conditioned on $X^{\left(1:M\right)}$, the representation *Z* carries no additional information about *Y* beyond what $X^{\left(1:M\right)}$ already provides [18]. By the data processing inequality, this gives $I\left(Z;Y\right)\leq I\left(X^{\left(1:M\right)};Y\right)$, and sufficiency (Definition 1) is the condition under which this bound is tight, equivalently $I(X^{\left(1:M\right)};Y|Z)=0$. Note that this characterization depends only on the encoder $q_{\phi }\left(z|x^{\left(1:M\right)}\right)$ and the true data distribution, not on any downstream predictor. A learned predictor $q_{\theta }\left(y|z\right)$ is a separate construct that approximates the Bayes-optimal rule p(y∣z); its quality affects practical performance but does not enter the definition of sufficiency.

**Definition 2**  (Minimal Sufficient Representation)**.** *A representation Z is minimal sufficient for predicting Y from $\left\{X^{\left(i\right)}\right\}$ if it is sufficient and minimizes $I(Z;X^{\left(1\right)},\ldots ,X^{\left(M\right)})$ over the class of sufficient representations.* 

Minimizing $I(Z;X^{\left(1:M\right)})$ encourages invariance to factors of variation irrelevant to prediction, such as scanner-specific effects, batch effects in omics data, or stylistic variation in clinical documentation [21,23]. A minimal sufficient representation thus captures biologically and clinically meaningful abstractions while discarding nuisance variability.

### 3.3. The Multimodal Information Bottleneck Objective

The trade-off between sufficiency and minimality is formalized by the multimodal information bottleneck (MIB) objective

(6) $$\min_{p(z|x^{\left(1:M\right)})}\;I\left(Z;X^{\left(1:M\right)}\right)-\beta \;I\left(Z;Y\right),$$

where β>0 controls the balance between compression and prediction. For sufficiently large β, solutions approach sufficiency; for smaller β, stronger compression is enforced, discarding information in $X^{\left(1:M\right)}$ that is irrelevant for *Y* [21]. This formulation is architecture-agnostic: convolutional networks, transformers, graph neural networks, and probabilistic latent-variable models can all be interpreted as approximate optimizers of (6) under different parameterizations.

### 3.4. Modality Structure and Information Allocation

Multimodality introduces structure absent in unimodal learning. Using the chain rule of mutual information, the total predictive information decomposes as

(7) $$I\left(X^{\left(1:M\right)};Y\right)=\sum_{i=1}^{M}I\left(X^{\left(i\right)};Y|X^{\left(1:i-1\right)}\right),$$

which highlights that the contribution of each modality is inherently context-dependent. Within the MIB framework, modality imbalance arises when *Z* allocates disproportionate capacity to a single dominant modality, retaining high $I(Z;X^{\left(i\right)})$ for some *i* while suppressing information from others. Such imbalance degrades robustness and can introduce fairness concerns in biomedical applications [11,12].

Representations with lower $I(Z;X^{\left(1:M\right)})$ are less sensitive to dataset-specific idiosyncrasies and are therefore more likely to generalize across institutions, acquisition protocols, and patient populations. When a subset S⊂{1,…,M} of modalities is observed, the achievable predictive information is limited by the lost conditional information $I(X^{\left(S^{c}\right)};Y|X^{\left(S\right)})$, where $S^{c}=\left\{1,\ldots ,M\right\}\backslash S$ denotes the missing modalities, establishing a direct link between information allocation and missing-data robustness that we formalize in Section 5.

### 3.5. Variational Bounds

The MIB objective (6) provides a principled formulation but is generally intractable for high-dimensional, continuous biomedical data. Both MI terms depend on unknown marginal distributions induced by the data-generating process and the encoder, and direct estimation in the dimensionality typical of biomedical modalities is impractical [23]. We therefore derive variational bounds that yield tractable learning objectives.

The compression term can be written as $I\left(Z;X^{\left(1:M\right)}\right)=E_{p^{⋆}\left(x^{\left(1:M\right)}\right)}\left[\mathrm{KL}\left(p\left(z|x^{\left(1:M\right)}\right)∥p\left(z\right)\right)\right]$, where $p\left(z\right)=\int p\left(z|x^{\left(1:M\right)}\right)\;p^{⋆}\left(x^{\left(1:M\right)}\right)\;dx^{\left(1:M\right)}$ is the (intractable) aggregate posterior. Introducing a variational prior $r_{\psi }\left(z\right)$ and exploiting the non-negativity of KL divergence yields the upper bound [23,55]

(8) $$I\left(Z;X^{\left(1:M\right)}\right)\leq E_{p^{⋆}\left(x^{\left(1:M\right)}\right)}\left[\mathrm{KL}\left(p\left(z|x^{\left(1:M\right)}\right)\;∥\;r_{\psi }\left(z\right)\right)\right].$$

The prior $r_{\psi }\left(z\right)$ may be a standard Gaussian, a mixture model, or a learned distribution such as a VampPrior [56].

For the predictive term, since I(Z;Y)=H(Y)−H(Y∣Z) and H(Y) is constant with respect to the encoder, maximizing I(Z;Y) is equivalent to minimizing H(Y∣Z). Introducing a variational decoder $q_{\theta }\left(y|z\right)$ gives the lower bound [23]

(9) $$I\left(Z;Y\right)\geq H\left(Y\right)+E_{p^{⋆}\left(x^{\left(1:M\right)},y\right)}\left[E_{p(z|x^{\left(1:M\right)})}\left[\log q_{\theta }\left(y|z\right)\right]\right].$$

Dropping the constant H(Y) recovers a standard expected negative log-likelihood objective, connecting the IB principle to supervised learning losses used in biomedical classification and regression [55].

### 3.6. The VMIB Objective

Combining (8) and (9) yields the variational multimodal information bottleneck (VMIB) objective

(10) $$\min_{\phi ,\theta ,\psi }\;L_{\mathrm{VMIB}}=\underset{\mathrm{predictive}\;\mathrm{loss}}{\underset{︸}{E_{p^{⋆}\left(x^{\left(1:M\right)},y\right)}\left[-\log q_{\theta }\left(y|z\right)\right]}}+\;\lambda \underset{\mathrm{compression}\;\mathrm{penalty}}{\underset{︸}{E_{p^{⋆}\left(x^{\left(1:M\right)}\right)}\left[\mathrm{KL}\left(q_{\phi }\left(z|x^{\left(1:M\right)}\right)\;∥\;r_{\psi }\left(z\right)\right)\right]}},$$

where λ=1/β controls the strength of compression and $q_{\phi }\left(z|x^{\left(1:M\right)}\right)$ is a parameterized multimodal encoder. The VMIB objective provides a direct information-theoretic interpretation of regularization in multimodal learning: the predictive loss encourages accuracy while the KL term enforces compression. Adjusting λ modulates the trade-off between performance and robustness, offering a principled mechanism for controlling overfitting in high-dimensional biomedical datasets. Importantly, the encoder $q_{\phi }$ may be implemented via modality-specific sub-encoders followed by attention-based or probabilistic fusion, transformer-based architectures, graph neural networks on biological interaction graphs, or hybrid probabilistic–neural designs; the VMIB constraints remain identical regardless of implementation [46].

The VMIB formulation recovers results from optimal IB theory [21] as special cases when the variational bounds are tight and the encoder family is sufficiently expressive. In realistic biomedical settings with continuous data, heterogeneous modalities, and finite samples, the variational approximation provides a practical and principled training objective.

Several IB variants offer complementary perspectives. The deterministic IB [30] restricts *Z* to be a deterministic function of *X*, yielding hard clustering solutions relevant to patient stratification tasks. The conditional entropy bottleneck [31] replaces I(Z;X) with H(Z∣Y), avoiding the need to estimate the marginal p(z) and often yielding tighter bounds. Goldfeld and Polyanskiy [32] provide a comprehensive analysis of IB solutions and their properties, including conditions under which the IB curve is convex. Kolchinsky et al. [33] demonstrate that, in deterministic settings, the compression term I(Z;X) can be infinite, requiring careful treatment through noise injection or geometric information measures. Wu et al. [34] analyze the learnability landscape of IB objectives, identifying phase transitions as β varies. These theoretical insights inform practical design choices for multimodal biomedical systems, where the choice of IB variant affects both the nature of learned representations and the tractability of optimization.

Contrastive learning provides an alternative route to information-optimal representations. The InfoNCE objective [25] maximizes a lower bound on I(Z;X) (or $I(Z^{\left(i\right)};Z^{\left(j\right)})$ across views), and has been extended to multiview settings through contrastive multiview coding [57]. CLIP-style models [27,58] align representations across modalities (e.g., images and text) via a contrastive loss that implicitly maximizes cross-modal MI while compressing modality-specific nuisance information through the limited dimensionality of the shared embedding space. The multi-view IB [46] formalizes this connection by deriving contrastive objectives as special cases of the IB Lagrangian under specific choices of variational families. In biomedical applications, this connection is practically important: contrastive pretraining on paired modalities (e.g., histopathology images and genomic profiles [7], or radiology images and clinical reports [59]) can be understood as implicit MIB optimization, with the contrastive temperature playing a role analogous to the IB trade-off parameter β.

**Figure 2:**
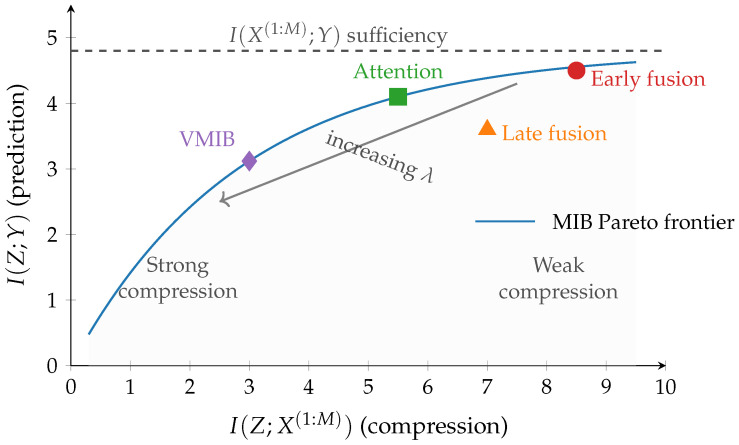
The multimodal information plane. Each point represents a learned representation *Z* characterized by its compression $I(Z;X^{\left(1:M\right)})$ and predictive information I(Z;Y). The MIB objective traces a blue Pareto frontier as β varies, while the dashed gray line marks the sufficiency ceiling $I(X^{\left(1:M\right)};Y)$. Points above the frontier are unachievable; the shaded region below it corresponds to suboptimal representations. The VMIB operates on the frontier at different compression levels. Existing architectures implicitly correspond to different operating points: early fusion retains most input information (weak compression), attention-based models achieve a favorable compression–prediction balance near the frontier, and late fusion falls below the frontier because independent per-modality compression discards joint information. Values and curves are illustrative.

Figure 2 illustrates the compression–prediction trade-off in the multimodal information plane.

### 3.7. Mapping Existing Architectures to the Framework

A key advantage of the MIB/VMIB formulation is its architecture-agnosticity: different multimodal architectures correspond to different implicit instantiations of the same information-theoretic objective. Table 2 makes this concrete by showing how prominent fusion strategies map onto the framework. Early fusion with a bottleneck layer directly approximates the MIB by reducing $I(Z;X^{\left(1:M\right)})$ through dimensional reduction while maximizing predictive accuracy. Late fusion corresponds to a factored MIB in which each modality is compressed independently. Attention-based transformers implement soft modality selection that dynamically adjusts the information retained from each source. Contrastive approaches such as CLIP-style models [58] achieve compression through low-dimensional projection and align representations across modalities via a contrastive objective rather than a supervised loss. Graph-based approaches such as MOGONET [8] operate on biological interaction graphs, performing compression through graph pooling while preserving topological predictive information. This mapping demonstrates that the MIB/VMIB framework provides a unifying language for comparing, analyzing, and improving diverse multimodal architectures on equal theoretical footing.

### 3.8. Synthetic Illustration

**Figure 3:**
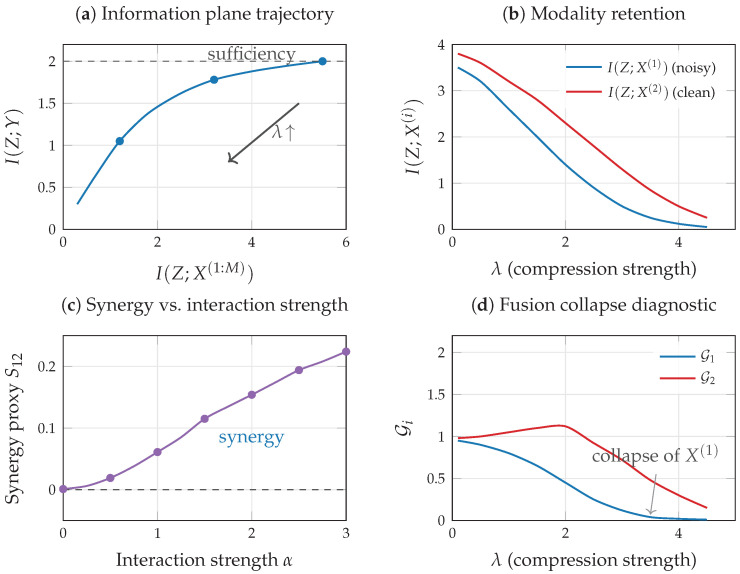
Synthetic two-modality illustration of the information-theoretic framework. (**a**) Schematic VMIB information-plane trajectory as compression λ increases; the dashed line marks the sufficiency ceiling $I(X^{\left(1\right)},X^{\left(2\right)};Y)$. (**b**) Schematic modality-wise information retention under increasing compression; the noisier modality ($X^{\left(1\right)}$) is compressed preferentially while the cleaner modality ($X^{\left(2\right)}$) is retained longer. (**c**) Computed synergy proxy $S_{12}$ as a function of interaction strength α in the model $Y|X∼\mathrm{Bernoulli}(\sigma \left(0.5\;X^{\left(1\right)}+0.5\;X^{\left(2\right)}+\alpha \;X^{\left(1\right)}X^{\left(2\right)}\right))$, estimated via a masked MLP on 5000 samples (mean over 5 seeds). $S_{12}$ is near zero at α=0 (additive) and increases monotonically as the interaction term creates genuine synergy. (**d**) Schematic fusion collapse diagnostics under noise imbalance: $G_{1}\to 0$ indicates that $X^{\left(1\right)}$ is effectively ignored, while $G_{2}$ initially dominates but ultimately decreases as overall I(Z;Y) declines at high compression. Both $G_{i}$ remain bounded above by I(Z;Y). Panels (**a**,**b**,**d**) are conceptual illustrations; panel (**c**) is computed from the stated model.

To make the framework concrete, consider a toy two-modality example with a genuinely synergistic generative process. Let $X^{\left(1\right)}∼N\left(0,1\right)$ and $X^{\left(2\right)}∼N\left(0,1\right)$ be two independent modalities (analogous to a gene expression measurement and a drug dosage), and let the binary target be generated as

$$Y|X^{\left(1\right)},X^{\left(2\right)}\;∼\;\mathrm{Bernoulli}\left(\sigma \left(\beta_{1}X^{\left(1\right)}+\beta_{2}X^{\left(2\right)}+\alpha \;X^{\left(1\right)}X^{\left(2\right)}\right)\right),$$

where σ is the logistic function and α≥0 controls the interaction strength. When α=0 (purely additive model), each modality provides independent predictive information about *Y*, and the synergy proxy $S_{12}=I\left(X^{\left(1\right)},X^{\left(2\right)};Y\right)-I\left(X^{\left(1\right)};Y\right)-I\left(X^{\left(2\right)};Y\right)\approx 0$. When α>0, the interaction term $\alpha \;X^{\left(1\right)}X^{\left(2\right)}$ creates predictive information that is accessible only through joint observation: neither $X^{\left(1\right)}$ nor $X^{\left(2\right)}$ alone captures the nonlinear interaction, so $S_{12}$ becomes positive and increases with α (Figure 3c, computed from 5000 training samples using a masked MLP with consistent MI estimation across subsets). This demonstrates that the synergy proxy can detect genuine interaction effects when modalities contribute through nonlinear combinations rather than additive signals.

Figure 3a,b,d illustrate the VMIB framework in a schematic two-modality scenario where one modality ($X^{\left(1\right)}$) is noisier than the other ($X^{\left(2\right)}$). Training a VMIB encoder with varying λ traces a curve in the information plane (Figure 3a). At small λ (weak compression), *Z* retains nearly all input information; as λ increases, *Z* progressively discards modality-specific noise, and modality retention $I(Z;X^{\left(i\right)})$ decreases in inverse proportion to predictive relevance (Figure 3b). The noisier modality is compressed preferentially, and if unchecked this can lead to fusion collapse with $G_{1}\approx 0$ (Figure 3d). These illustrations demonstrate how the quantities defined in this section and in Section 4 can be monitored during training and used to diagnose representation quality.

## 4. Decomposing Information Across Modalities

A central challenge in multimodal learning is understanding how predictive information about *Y* is distributed across modalities. Empirical gains from fusion can arise from fundamentally different mechanisms (redundancy reduction, complementary information integration, or synergistic interactions), and distinguishing these requires principled analysis. This section develops an information-theoretic decomposition framework that operates both at the input level and within learned representations.

### 4.1. Unimodal and Incremental Contributions

For a given modality $X^{\left(i\right)}$, the unimodal predictive information $I(X^{\left(i\right)};Y)$ measures the reduction in uncertainty about *Y* achieved by observing $X^{\left(i\right)}$ alone and provides a modality-specific baseline for achievable performance. Critically, the high entropy or dimensionality of a modality does not imply high predictive information: raw imaging data may exhibit large $H(X^{\left(i\right)})$ due to pixel-level variability while containing relatively little task-relevant information [18].

The incremental contribution of $X^{\left(i\right)}$ given a subset S⊆{1,…,M} of other modalities is captured by the conditional mutual information $I(X^{\left(i\right)};Y|X^{\left(S\right)})$, which quantifies how much additional predictive information $X^{\left(i\right)}$ provides beyond what is already available from $X^{\left(S\right)}$. In biomedical contexts, this quantity clarifies whether genomic profiles add meaningful insight once imaging features are known, or whether wearable signals contribute beyond routinely collected clinical variables.

### 4.2. Redundancy, Synergy, and Their Relationship to Partial Information Decomposition

Multimodal gains may arise from two distinct mechanisms. Redundancy occurs when modalities encode overlapping predictive information about *Y*, while synergy arises when predictive information becomes accessible only through joint observation. The partial information decomposition (PID) framework [24] provides a rigorous decomposition of $I(X^{\left(i\right)},X^{\left(j\right)};Y)$ into unique, redundant, and synergistic components. However, the PID requires specifying a redundancy measure whose choice remains debated [60], and exact computation is intractable for high-dimensional continuous variables typical of biomedical data.

We therefore adopt operationally motivated proxies that are tractable and interpretable in practice. For two modalities $X^{\left(i\right)}$ and $X^{\left(j\right)}$, we define the redundancy proxy

(11) $$R_{ij}≜I\left(X^{\left(i\right)};Y\right)+I\left(X^{\left(j\right)};Y\right)-I\left(X^{\left(i\right)},X^{\left(j\right)};Y\right),$$

which by the inclusion-exclusion identity equals the mutual information $I(X^{\left(i\right)};X^{\left(j\right)};Y)$ in the co-information sense [61,62]. When $R_{ij}>0$, the individual modalities together over-count predictive information, indicating overlap. The synergy proxy is defined as

(12) $$S_{ij}≜I\left(X^{\left(i\right)},X^{\left(j\right)};Y\right)-I\left(X^{\left(i\right)};Y\right)-I\left(X^{\left(j\right)};Y\right)=-R_{ij}.$$

**Figure 4:**
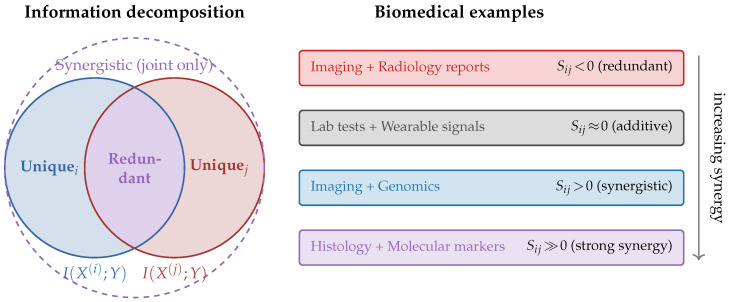
Illustration of the redundancy–synergy decomposition for two biomedical modalities. (**Left**): Venn diagram showing unique, redundant, and synergistic information components; the dashed outer boundary represents synergistic information accessible only through joint observation. (**Right**): representative biomedical modality pairs along the redundancy–synergy spectrum. Negative $S_{ij}$ (redundancy-dominated) indicates overlapping predictive content where fusion gains arise primarily from noise averaging; imaging paired with radiology reports is a canonical example, as both describe the same visual findings. Positive $S_{ij}$ (synergy-dominated) indicates that joint observation unlocks predictive information inaccessible to either modality alone; histology combined with molecular markers exhibits strong synergy because nonlinear interactions between morphological phenotypes and molecular signatures reveal disease mechanisms invisible to single-modality analysis.

By construction, $S_{ij}>0$ indicates that the joint observation of modalities yields strictly more predictive information than the sum of individual contributions, a phenomenon observed in precision medicine where imaging phenotypes and molecular signatures interact nonlinearly [2,7]. When $S_{ij}<0$ (equivalently $R_{ij}>0$), redundancy dominates: the modalities share overlapping predictive content, suggesting that fusion gains arise primarily from noise averaging rather than complementary signal integration. The case $S_{ij}\approx 0$ indicates approximate additivity. We emphasize that these proxies conflate redundancy and synergy into a single axis and do not recover the full four-way PID decomposition; they should be interpreted as net indicators of interaction type rather than exact measurements of each component [24,60]. Figure 4 illustrates the redundancy–synergy spectrum for representative biomedical modality pairs.

### 4.3. Decomposition in the Learned Representation Space

The preceding analysis applies to raw inputs. In learned systems, predictions are made from latent representations *Z*, and a key question is how information is allocated after fusion. The quantity $I(Z;X^{\left(i\right)})$ measures how much information about modality *i* is retained in the representation. A well-balanced representation should allocate capacity in proportion to predictive relevance; disproportionate values of $I(Z;X^{\left(i\right)})$ across modalities signal over-reliance on a single data source.

### 4.4. Connections to Explainability and Feature Attribution

Information decomposition connects naturally to explainability methods in multimodal biomedical ML. The conditional mutual information $I(X^{\left(i\right)};Y|X^{\left(S\right)})$ quantifies the unique contribution of modality *i* beyond a reference set *S*, which is closely related to Shapley-value-based feature attribution [63,64]. Specifically, the Shapley value of modality *i* is a weighted average of $I(X^{\left(i\right)};Y|X^{\left(S\right)})$ over all subsets S⊆{1,…,M}∖{i}, providing a fair allocation of predictive credit that accounts for all possible modality interactions [64]. In the biomedical context, this enables clinicians to understand not only which modalities are most informative for a given prediction but also whether the value of a modality arises from its unique content, its redundancy with other sources (providing robustness), or its synergistic interactions (providing emergent predictive power). For instance, Steyaert et al. [41] demonstrated that information-theoretic attribution can reveal complementary roles of transcriptomic and histological features in cancer prognosis that would be invisible to single-modality analyses.

### 4.5. Diagnosing Fusion Collapse

A common failure mode in multimodal learning is fusion collapse, in which the learned representation effectively ignores one or more modalities [11,12]. We formalize this through the modality-conditioned predictive gap

(13) $$G_{i}≜I\left(Z;Y\right)-I\left(Z;Y|X^{\left(i\right)}\right).$$

By the chain rule, $G_{i}=I\left(Z;X^{\left(i\right)}\right)-I\left(Z;X^{\left(i\right)}|Y\right)$, so $G_{i}$ captures the portion of information that *Z* retains about $X^{\left(i\right)}$ that is predictively relevant. If $G_{i}\approx I\left(Z;Y\right)$ for a single modality *i* while $G_{j}\approx 0$ for j≠i, the representation is dominated by modality *i*. This diagnostic is more informative than simply monitoring $I(Z;X^{\left(i\right)})$, which conflates predictive and nuisance information.

**Figure 5:**
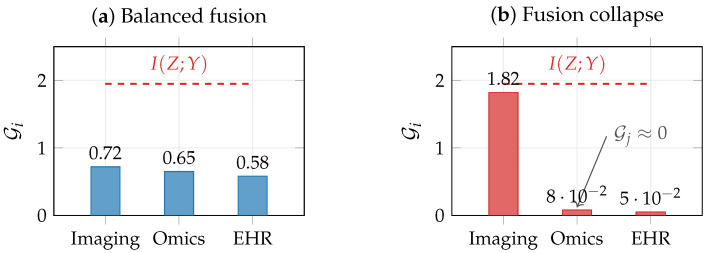
Diagnosing fusion collapse via modality-conditioned predictive gaps $G_{i}$. (**a**) A balanced representation distributes predictive reliance across modalities, with $G_{i}$ comparable for all *i* and each well below I(Z;Y). (**b**) Fusion collapse concentrates predictive reliance in a single dominant source: $G_{\mathrm{Imaging}}\approx I\left(Z;Y\right)$ while $G_{\mathrm{Omics}}$ and $G_{\mathrm{EHR}}$ are near zero, indicating that the representation effectively ignores these modalities. The dashed line indicates the total predictive information I(Z;Y), which upper-bounds every individual $G_{i}$ by definition (since $G_{i}=I\left(Z;Y\right)-I\left(Z;Y|X^{\left(i\right)}\right)$ and $I(Z;Y|X^{\left(i\right)})\geq 0$). In clinical settings, fusion collapse leads to brittle predictions that degrade sharply when the dominant modality is unavailable or corrupted. Values and curves are illustrative.

We note an important limitation: $G_{i}$ is a co-information quantity that is most interpretable in redundancy-dominated settings. In synergy-dominated tasks where predictive information arises only through joint observation (e.g., interaction effects), a modality can be essential yet have $G_{i}\approx 0$ because it provides no marginal predictive value. In such settings, ablation-based Shapley values or partial information decomposition may provide more appropriate diagnostics [24,64]. In biomedical applications, where redundancy among correlated data sources is common, fusion collapse leads to brittle models that perform well only when the dominant modality is present, undermining clinical reliability. Figure 5 illustrates balanced versus collapsed multimodal representations.

## 5. Missing-Modality Robustness as Information Consistency

Missing or partially observed modalities are a defining characteristic of real-world biomedical data [3,5]. Clinical records are often incomplete, imaging may be contraindicated, and wearable data streams may be interrupted. A central requirement for multimodal biomedical ML is graceful degradation: predictive performance should deteriorate predictably as information is removed, rather than failing catastrophically.

### 5.1. Missing Modalities as Information Loss

Let S⊆{1,…,M} index the observed modalities and $S^{c}$ its complement. The maximum achievable mutual information $I(Z^{\left(S\right)};Y)$ under partial observation is limited by the conditional information loss

(14) $$I_{\mathrm{miss}}\left(S\right)≜I\left(X^{\left(S^{c}\right)};Y|X^{\left(S\right)}\right),$$

which measures the predictive information about *Y* that is inaccessible when modalities in $S^{c}$ are absent. This quantity is independent of the learning algorithm and reflects an intrinsic property of the data-generating process [18]. Not all missing modalities are equally consequential: the absence of a modality with low $I(X^{\left(i\right)};Y|X^{\left(S\right)})$ has negligible impact, while the absence of a highly informative modality imposes unavoidable performance degradation.

Existing approaches to handling missing modalities fall into three broad categories. Imputation-based methods attempt to reconstruct missing data from observed modalities, for example through cross-modal autoencoders or generative models; however, the data processing inequality guarantees that imputed modalities cannot contain more predictive information than the observed data that generated them, placing a fundamental limit on imputation quality. Architecture-based methods design models that accept variable-size inputs through masking, set functions, or modular encoder designs [65]; these approaches are practical but typically lack formal guarantees about degradation behavior. Regularization-based methods encourage robustness during training through modality dropout, where random subsets of modalities are zeroed out during training, an approach that can be viewed as a Monte Carlo approximation to the consistency objective developed below.

### 5.2. Information Consistency and the Augmented VMIB

Let $Z^{\left(S\right)}∼q_{\phi }\left(z|x^{\left(S\right)}\right)$ denote the representation inferred from partial observations and $Z^{\left(1:M\right)}∼q_{\phi }\left(z|x^{\left(1:M\right)}\right)$ the full-information representation. The key principle is *information consistency*: representations inferred from different modality subsets should agree to the extent permitted by available information. We note that this formulation requires the encoder $q_{\phi }$ to accept variable-cardinality inputs, which can be realized through modality-specific sub-encoders with a shared fusion module, masking mechanisms, or set-function architectures [46,65].

A robust representation should satisfy $I\left(Z^{\left(S\right)};Y\right)\approx I\left(Z^{\left(1:M\right)};Y\right)-I_{\mathrm{miss}}\left(S\right)$, meaning that performance degradation reflects the true loss of predictive information rather than representational failure. We enforce this through a KL-based consistency penalty, yielding the augmented objective

(15) $$\min_{\phi ,\theta ,\psi }\;L=L_{\mathrm{VMIB}}+\gamma \sum_{S\in S}E_{p^{⋆}\left(x^{\left(1:M\right)}\right)}\left[\mathrm{KL}\left(q_{\phi }\left(z|x^{\left(1:M\right)}\right)\;∥\;q_{\phi }\left(z|x^{\left(S\right)}\right)\right)\right],$$

where S is a collection of modality subsets of interest and γ>0 controls the consistency strength. The choice of KL direction in (15) is deliberate: placing the full-information posterior $q_{\phi }\left(z|x^{\left(1:M\right)}\right)$ as the first argument yields a forward KL that encourages the partial-observation encoder $q_{\phi }\left(z|x^{\left(S\right)}\right)$ to cover the support of the full encoder (mean-seeking behavior), rather than collapsing onto a single mode as the reverse KL would encourage. This is preferable in practice because the partial encoder should represent uncertainty about the missing information rather than commit to a point estimate. This formulation is architecture-agnostic, provides a principled alternative to heuristic data imputation or modality dropout strategies [65], and explicitly ties robustness to an information-theoretic objective ensuring interpretable and predictable degradation.

**Figure 6:**
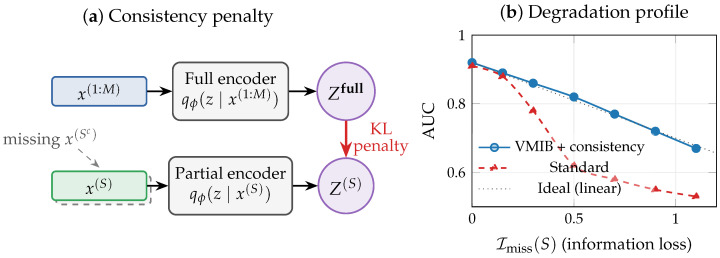
Missing-modality robustness as information consistency. (**a**) The forward KL penalty $\mathrm{KL}(q_{\phi }\left(z|x^{\left(1:M\right)}\right)∥q_{\phi }\left(z|x^{\left(S\right)}\right))$ aligns partial-observation representations $Z^{\left(S\right)}$ with the full-information representation $Z^{\mathrm{full}}$; the unidirectional arrow reflects the asymmetry of KL divergence, which encourages the partial encoder to cover the support of the full encoder (mean-seeking behavior) rather than collapsing onto a single mode. (**b**) Models trained with the consistency penalty (blue, solid) exhibit graceful, near-linear degradation that closely tracks the ideal profile (dotted), where performance loss is proportional to the information loss $I_{\mathrm{miss}}\left(S\right)$. Standard models (red, dashed) degrade steeply and erratically, with AUC dropping far below the ideal profile even at moderate levels of missingness. Values and curves are illustrative.

At test time, predictions are made using $Z^{\left(S\right)}$ when only a subset of modalities is available. The expected performance degradation $\Delta_{\mathrm{miss}}\left(S\right)=E\left[\ell \left(\hat{Y}\left(Z^{\left(S\right)}\right),Y\right)-\ell \left(\hat{Y}\left(Z^{\left(1:M\right)}\right),Y\right)\right]$ is expected to correlate with $I_{\mathrm{miss}}\left(S\right)$ under the consistency framework, though the precise relationship depends on the loss function (the link is exact for log-loss via the chain rule of MI, and heuristic for rank-based metrics such as AUC). This design principle enables clinicians to anticipate model performance under specific missing-data patterns and supports informed decisions about data acquisition priorities. Figure 6 illustrates the information consistency principle.

## 6. Uncertainty, Calibration, and Trust

Trustworthy biomedical ML systems must quantify uncertainty in a principled manner and produce reliable probability estimates across heterogeneous deployment conditions [13,15]. We develop an information-theoretic treatment of uncertainty and calibration within the VMIB framework.

### 6.1. Predictive Uncertainty and Its Decomposition

Let ${\hat{p}}_{\theta }\left(y|z\right)$ denote a probabilistic predictor. The predictive entropy

(16) $$H\left({\hat{p}}_{\theta }\left(\cdot |z\right)\right)=-\sum_{y\in Y}{\hat{p}}_{\theta }\left(y|z\right)\log{\hat{p}}_{\theta }\left(y|z\right)$$

quantifies the uncertainty of the model’s output for a given input. From an information-theoretic perspective, the cross-entropy $E_{p(y|z)}\left[-\log{\hat{p}}_{\theta }\left(y|z\right)\right]$ upper-bounds H(Y∣Z) by Gibbs’ inequality and coincides with it when the predictor is Bayes-optimal [18]. The nonnegative cross-entropy gap

(17) $$E_{p(z)}\left[\mathrm{KL}\left(p\left(\cdot |z\right)\;∥\;{\hat{p}}_{\theta }\left(\cdot |z\right)\right)\right]=E\left[-\log{\hat{p}}_{\theta }\left(Y|Z\right)\right]-H\left(Y|Z\right)\;\geq \;0$$

measures model mismatch between the true and predicted conditional distributions. We caution that the predictive entropy $H({\hat{p}}_{\theta }\left(\cdot |z\right))$ is not guaranteed to exceed H(Y∣Z): an overconfident model may produce $H({\hat{p}}_{\theta }\left(\cdot |z\right))<H\left(Y|Z\right)$, making predictive entropy alone an unreliable indicator of total uncertainty. The nonnegative quantity in Equation (17) provides the theoretically grounded measure.

Uncertainty in biomedical prediction can be decomposed into aleatoric and epistemic components [66]. From an information-theoretic perspective, the aleatoric component corresponds to the irreducible conditional entropy H(Y∣Z), reflecting biological and measurement noise. The cross-entropy gap of Equation (17) captures model mismatch, which is related to but not identical with epistemic uncertainty: the gap reflects discrepancy between the learned and true conditionals, whereas epistemic uncertainty in the Bayesian sense additionally requires a posterior over model parameters. However, we emphasize that practical separation of these components requires additional modeling assumptions beyond the information-theoretic framing alone, such as ensemble disagreement [67], Monte Carlo dropout [68], or Bayesian posterior inference [66]. Within the IB framework, increasing I(Z;Y) reduces H(Y∣Z) and thus lowers the aleatoric floor. The information-theoretic perspective clarifies the fundamental limits ($H(Y|X^{\left(1:M\right)})$ is irreducible regardless of method) while leaving the choice of epistemic uncertainty estimator to the practitioner.

### 6.2. Distribution Shift and OOD Detection

Under distribution shift from $p_{\mathrm{train}}$ to $p_{\mathrm{test}}$, learned representations may encode less predictive information, leading to $I_{p_{\mathrm{test}}}\left(Z;Y\right)<I_{p_{\mathrm{train}}}\left(Z;Y\right)$ and consequently higher predictive entropy. This motivates entropy-based out-of-distribution (OOD) detection: samples with $H({\hat{p}}_{\theta }\left(\cdot |z\right))>\tau$ for a chosen threshold τ are flagged as potentially unreliable [69]. Entropy thresholding remains an operational heuristic, but it is naturally motivated by the possibility that distribution shift reduces predictive information and increases uncertainty within the information-optimization framework.

### 6.3. Calibration

A well-calibrated model satisfies $P(Y=1|\hat{P}=p)=p$ for all p∈[0,1], where $\hat{P}={\hat{p}}_{\theta }\left(Y=1|Z\right)$ [70]. Calibration quality is typically assessed via the expected calibration error (ECE), reliability diagrams, or proper scoring rules such as the Brier score. From an information-theoretic perspective, the conditional entropy $H(Y|\hat{P})$ captures the resolution (sharpness) of the predictive distribution, though resolution and calibration are distinct properties in the proper-score decomposition. In multimodal settings, calibration errors often arise from modality domination: if *Z* over-relies on a dominant modality that shifts across cohorts, predictive probabilities become systematically miscalibrated. Monitoring $I(Z;X^{\left(i\right)})$ and the modality-conditioned predictive gap $G_{i}$ thus provides early warning of calibration failure [11,70].

### 6.4. Risk-Sensitive Decision Making

Clinical decisions often involve asymmetric costs. Entropy-based uncertainty naturally supports risk-sensitive policies through a deferral rule

(18) $$\delta \left(z\right)=\left\{\begin{array}{cc} \mathrm{predict}, & H\left({\hat{p}}_{\theta }\left(\cdot |z\right)\right)\leq \tau ,\\ \mathrm{defer}\;\mathrm{to}\;\mathrm{clinician}, & \mathrm{otherwise}, \end{array}\right.$$

which escalates high-entropy cases to human experts. Such policies align with clinical practice by explicitly linking uncertainty to action [13,15].

### 6.5. Conformal Prediction and Distribution-Free Guarantees

While entropy-based uncertainty provides useful heuristic signals, it does not in general offer formal coverage guarantees. Conformal prediction [71] offers a complementary, distribution-free framework: given a nonconformity score (which may be derived from predictive entropy or other model outputs), conformal methods construct prediction sets C(z)⊆Y satisfying P(Y∈C(Z))≥1−α for a user-specified error rate α, under only the assumption of exchangeable data [72]. In multimodal biomedical settings, the size of the conformal prediction set |C(z)| serves as an interpretable measure of uncertainty: well-calibrated multimodal models should produce smaller prediction sets (higher precision) than unimodal baselines, reflecting the additional information captured by fusion. Conversely, when modalities are missing, conformal set sizes should expand in proportion to $I_{\mathrm{miss}}\left(S\right)$, providing a distribution-free counterpart to the information-theoretic degradation bounds of Section 5. The intersection of conformal methods with information-theoretic principles, for instance using MI-based nonconformity scores or information-aware calibration procedures, represents a promising direction for achieving both principled uncertainty quantification and formal statistical guarantees in clinical AI systems.

**Figure 7:**
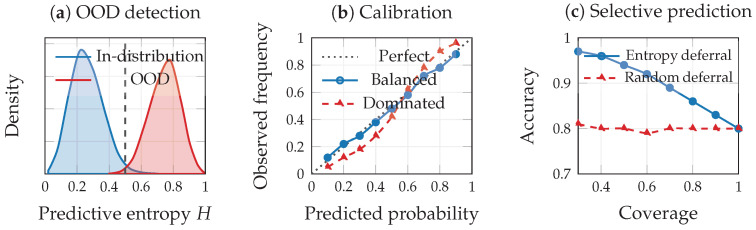
Entropy-based uncertainty and calibration diagnostics. (**a**) Predictive entropy separates in-distribution samples (low entropy, blue) from out-of-distribution samples (high entropy, red), enabling threshold-based OOD detection at threshold τ; the overlap region reflects ambiguous cases near the decision boundary. (**b**) Reliability diagram: the dotted diagonal represents perfect calibration; the balanced multimodal model (blue, solid) tracks it closely, while the modality-dominated model (red, dashed) exhibits systematic miscalibration, being overconfident at low predicted probabilities and underconfident at high ones. (**c**) Selective prediction via entropy-based deferral (blue, solid) yields substantial accuracy gains on retained predictions by routing uncertain cases to human experts; random deferral (red, dashed) provides no systematic improvement, confirming that the accuracy gains are attributable to the informativeness of entropy as an uncertainty measure rather than to selection effects. Values and curves are illustrative.

Figure 7 illustrates how entropy-based diagnostics support trust.

## 7. Longitudinal Disease Modeling and Information Flow

Many biomedical phenomena unfold over time: disease progression, treatment response, and recovery involve complex temporal interactions [2,5]. We extend the framework to longitudinal settings by viewing disease modeling as an information flow problem.

### 7.1. Sequential Information Bottleneck

Let $\left\{X_{t}^{\left(1\right)},\ldots ,X_{t}^{\left(M\right)}\right\}$ denote multimodal observations at time t=1,…,T and $Y_{t}$ a disease-related target. A longitudinal model constructs a sequence of latent representations $Z_{t}∼p\left(z_{t}|X_{1:t}^{\left(1:M\right)},Z_{1:t-1}\right)$ with predictions via $p(y_{t}|z_{t})$. The representation $Z_{t}$ serves as a compressed summary of the patient’s history up to time *t*.

The temporal extension of the IB principle yields the sequential information bottleneck (SIB) objective

(19) $$\min_{p(z_{t}|\cdot )}\;I\left(Z_{t};X_{1:t}^{\left(1:M\right)}\right)-\beta \;I\left(Z_{t};Y_{t:t+\tau }\right),$$

where τ≥0 is the prediction horizon. This objective ensures that $Z_{t}$ retains information predictive of future disease evolution while compressing the growing historical record, mitigating noise accumulation and preventing overfitting to idiosyncratic temporal patterns [21,23]. Recurrent neural networks, state-space models, and temporal transformers can all be interpreted as approximate solutions to (19), differing in how they parameterize temporal dependencies.

### 7.2. Transfer Entropy for Temporal Modality Analysis

The transfer entropy from modality $X^{\left(i\right)}$ to disease variable *Y*, defined as

(20) $${\mathrm{TE}}_{X^{\left(i\right)}\to Y}\left(t\right)=I\left(X_{t-k:t-1}^{\left(i\right)};\;Y_{t}|Y_{t-\ell :t-1}\right),$$

where *k* and *ℓ* denote the lag orders for the source modality and target variable, respectively, measures the additional predictive information provided by the recent history of modality *i* beyond what is explained by *Y*’s own past. Tracking ${\mathrm{TE}}_{X^{\left(i\right)}\to Y}\left(t\right)$ over time provides a principled mechanism for temporal modality prioritization: modalities with persistently low transfer entropy may be sampled less frequently, while those with increasing influence warrant closer monitoring [53,54]. At the representation level, the transfer entropy ${\mathrm{TE}}_{Z\to Y}=I\left(Z_{t-1};Y_{t}|Y_{t-1}\right)$ measures how well the latent state captures predictive dynamics; a decline over time may signal representation drift or model degradation.

**Figure 8:**
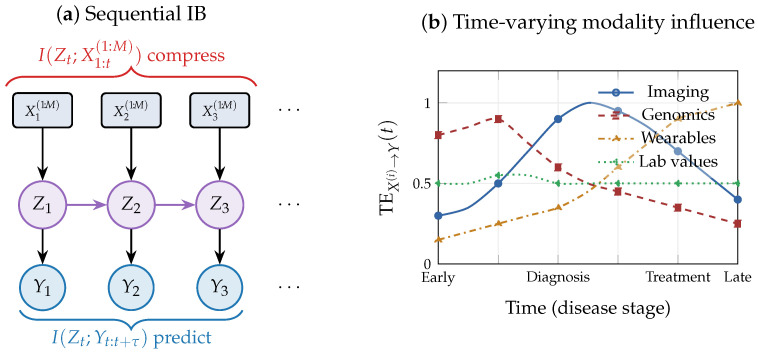
Longitudinal disease modeling as information flow. (**a**) The sequential information bottleneck constructs latent representations $Z_{t}$ that compress the growing patient history $X_{1:t}^{\left(1:M\right)}$ (red brace) while preserving information predictive of future disease outcomes $Y_{t:t+\tau }$ (blue brace); purple arrows indicate temporal state transitions. (**b**) Transfer entropy ${\mathrm{TE}}_{X^{\left(i\right)}\to Y}\left(t\right)$ reveals time-varying modality influence across the disease course: genomic data (dashed) provides the strongest predictive signal in early stages when inherited risk factors dominate; imaging (solid) peaks around diagnosis and staging; wearable signals (dash-dotted) become increasingly informative during treatment monitoring and follow-up; and lab values (dotted) provide approximately constant moderate information throughout. These profiles suggest that adaptive data acquisition (prioritizing different modalities at different disease stages) can improve both efficiency and predictive performance. Values and curves are illustrative.

Longitudinal prediction compounds uncertainty. The conditional entropy $H(Y_{t}|Z_{t-1})$ quantifies uncertainty in the current disease state given past representations. Effective longitudinal models should reduce this quantity as new observations arrive while gracefully increasing it when observations are sparse. Coupling SIB objectives with entropy-based uncertainty tracking thus supports early warning systems and timely clinical intervention. Figure 8 illustrates the longitudinal information flow framework.

## 8. Implications for Multimodal Foundation Models

Multimodal foundation models (MFMs) have emerged as a dominant paradigm in biomedical AI, leveraging large-scale pretraining across heterogeneous data sources [9,58,73]. Notable examples include BiomedCLIP [58], which aligns biomedical images with text descriptions via contrastive pretraining on 15 million scientific image–text pairs; Med-PaLM [74], a large language model achieving expert-level performance on medical question answering; PLIP [59], a pathology-specific vision–language model; and emerging generalist systems such as Med-Gemini [75] that integrate imaging, genomics, and clinical text. While achieving impressive performance, their scale obscures fundamental questions about representation quality, modality utilization, and robustness. The information-theoretic framework provides principled, architecture-agnostic diagnostics.

### 8.1. Foundation Models as Multi-Task Information Compressors

An MFM maps inputs $X^{\left(1:M\right)}$ to a representation $Z_{\theta }∼p_{\theta }\left(z|x^{\left(1:M\right)}\right)$ pretrained on a mixture of objectives. This can be viewed as optimizing a multi-task generalization of the IB

(21) $$\min_{\theta }\;I\left(Z_{\theta };X^{\left(1:M\right)}\right)-\sum_{k}\beta_{k}\;I\left(Z_{\theta };Y^{\left(k\right)}\right),$$

where $\left\{Y^{\left(k\right)}\right\}$ denote downstream or proxy tasks and the task-specific weights $\beta_{k}$ control the relative importance of each objective [73,76]. By constraining $I(Z_{\theta };X^{\left(1:M\right)})$, pretraining discourages memorization of dataset-specific artifacts while promoting reusable abstractions, explaining the strong transfer performance of MFMs. The multi-task formulation introduces a tension between breadth (preserving information relevant to many tasks) and specificity (compressing aggressively for a single downstream target), which adaptation must resolve.

### 8.2. Information-Theoretic Diagnostics and Adaptation

The diagnostics developed in Section 4 (representation entropy $H(Z_{\theta })$, modality retention $I(Z_{\theta };X^{\left(i\right)})$, and the fusion collapse diagnostic $G_{i}$) apply directly to MFMs, providing a compact audit of representation quality independent of architectural details [23]. We focus here on the two aspects unique to the foundation model setting.

First, information-efficient adaptation: foundation models are typically adapted to downstream tasks via fine-tuning or lightweight adapters [77]. Let $Z_{\theta }$ denote the pretrained and $Z_{\theta^{\prime }}$ the adapted representation. An information-efficient adaptation satisfies the joint condition that $I\left(Z_{\theta^{\prime }};Y\right)-I\left(Z_{\theta };Y\right)$ is large (high predictive gain) while $I\left(Z_{\theta^{\prime }};X^{\left(1:M\right)}\right)-I\left(Z_{\theta };X^{\left(1:M\right)}\right)$ is small (minimal increase in input sensitivity) [31]. This formalizes the intuition that adaptation should add task-relevant information without reintroducing nuisance variability, and motivates entropy-aware regularization during fine-tuning, particularly in low-label biomedical regimes.

**Figure 9:**
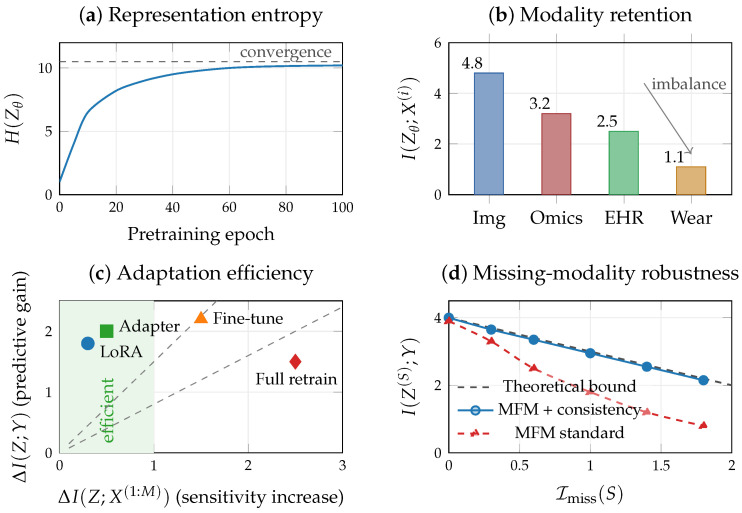
Information-theoretic diagnostic dashboard for multimodal foundation models. (**a**) Representation entropy $H(Z_{\theta })$ increases during pretraining as the model learns richer representations, converging toward a plateau that reflects the effective dimensionality of the learned embedding space. (**b**) Modality retention profile $I(Z_{\theta };X^{\left(i\right)})$ reveals potential imbalance: imaging dominates the representation while wearable data are under-represented, indicating possible fusion collapse detectable via the $G_{i}$ diagnostic of Section 4. (**c**) Adaptation strategies plotted by predictive gain versus sensitivity increase: lightweight methods (LoRA, adapters) achieve high predictive gain with minimal sensitivity increase (efficiency ratios of 6.0 and 4.0, respectively), while full retraining incurs large sensitivity increases with diminishing returns (ratio 0.6), consistent with catastrophic forgetting of pretrained abstractions. Dashed lines indicate iso-efficiency contours. (**d**) Missing-modality robustness: models trained with the consistency penalty (blue, solid) degrade gracefully, tracking within 0.05 of the theoretical information-loss bound at all levels of missingness; standard models (red, dashed) degrade far below the bound, losing substantially more predictive information than the missing data warrants. Values and curves are illustrative.

Second, robustness under partial observation: the missing-modality consistency framework of Section 5 extends naturally to MFMs. Robust models should satisfy $\mathrm{KL}(p_{\theta }\left(z|x^{\left(1:M\right)}\right)∥p_{\theta }\left(z|x^{\left(S\right)}\right))$ small, up to the intrinsic loss $I(X^{\left(S^{c}\right)};Y|X^{\left(S\right)})$, while predictive entropy $H({\hat{p}}_{\theta }\left(\cdot |Z_{\theta }\right))$ serves as a scalable uncertainty measure aligned with the IB objective. Figure 9 summarizes the diagnostic framework for MFMs.

## 9. Empirical Validation

The preceding sections developed an information-theoretic framework through definitions, propositions, and a synthetic illustration. To illustrate that the framework’s key quantities are computable, interpretable, and practically useful on real biomedical data, we present empirical case studies across three datasets: TCGA breast cancer multi-omics (information decomposition, compression–prediction tradeoff, missing-modality robustness, fusion collapse), TCGA glioma clinical-plus-molecular data (cross-level modality contrast), and OASIS-2 longitudinal Alzheimer’s data (transfer entropy, sequential prediction). We additionally evaluate uncertainty quantification and selective prediction on BRCA, and pretraining/adaptation diagnostics in a controlled setting.

### 9.1. Dataset and Experimental Setup

We use the processed TCGA-BRCA dataset from Wang et al. [8], comprising three omics modalities for N=875 breast cancer patients (612 training, 263 test): (i) mRNA expression ($d_{1}=1000$ features), (ii) DNA methylation ($d_{2}=1000$ features), and (iii) miRNA expression ($d_{3}=503$ features). The prediction target *Y* is a 5-class PAM50 molecular subtype classification (Basal-like, HER2-enriched, Luminal A, Luminal B, and Normal-like) [78], with entropy H(Y)=1.352 nats reflecting the imbalanced class distribution.

All features are standardized to zero mean and unit variance. The VMIB model uses per-modality encoders (two-layer MLPs with 256 hidden units, batch normalization, and dropout p=0.3), concatenation-based fusion, a variational bottleneck with $d_{Z}=32$ latent dimensions and standard Gaussian prior, and a two-layer predictor. Models are trained with Adam (learning rate $10^{-3}$, cosine annealing) for 100–150 epochs on an NVIDIA GPU using PyTorch 2.10.0+cu126. We report means over three random seeds where noted.

Mutual information lower bounds are estimated via the classification approach: I(X;Y)≥H(Y)+E[logq(y∣x)], where *q* is a trained MLP classifier [29]. Separate classifiers are trained for each modality subset with early stopping on a validation split (80/20 stratified within the training set). The VMIB objective directly provides the compression upper bound $I\left(Z;X^{\left(1:M\right)}\right)\leq \mathrm{KL}\left(q_{\phi }\left(z|x^{\left(1:M\right)}\right)∥r_{\psi }\left(z\right)\right)$ and the prediction lower bound I(Z;Y)≥H(Y)−CE(Y∣Z).

Estimation caveats.

All MI estimates are lower bounds whose tightness depends on the classifier’s capacity and training quality. Because separate classifiers are used for each modality subset, bound quality may vary with input dimensionality: higher-dimensional inputs (modality combinations) may yield looser bounds, and the estimated MI for a superset can fall below that of a subset. Differences of lower bounds are not lower bounds on differences; accordingly, synergy proxies computed as $S_{ij}=\hat{I}\left(X^{\left(i\right)},X^{\left(j\right)};Y\right)-\hat{I}\left(X^{\left(i\right)};Y\right)-\hat{I}\left(X^{\left(j\right)};Y\right)$ should be interpreted as indicative of the direction and relative magnitude of modality interactions rather than as exact quantities. We report values averaged over 5-fold stratified cross-validation. Additionally, MI bounds constrain achievable error rates via Fano’s inequality, but AUC is a rank-based metric without a direct MI bound; the empirical correspondence between $I_{\mathrm{miss}}$ and AUC degradation observed below serves as an informative reference rather than a theoretical guarantee.

### 9.2. Information Decomposition Across Modalities

Table 3 reports estimated MI between each modality (and combination) and the target. Among individual modalities, mRNA expression provides the highest predictive information ($I(X^{\left(1\right)};Y)\geq 0.878\pm 0.044$ nats, capturing 64.9% of H(Y)), consistent with mRNA’s central role in defining molecular subtypes. DNA methylation and miRNA contribute lower but substantial fractions (48.4% and 46.9% of H(Y), respectively).

All three pairwise synergy proxies are negative ($S_{ij}<0$) across all five CV folds. We emphasize that $S_{ij}$, computed from differences of separate MI lower bounds, should be interpreted as a heuristic interaction proxy rather than a rigorous measurement of redundancy or synergy: the sign may reflect genuine modality overlap, but could also be influenced by differential bound tightness across input dimensions (see estimation caveats above). With this caveat, the consistently negative sign is at least consistent with redundancy among same-level omics modalities, as biologically expected: mRNA, methylation, and miRNA all reflect aspects of the same underlying transcriptional programs [78]. A 5-fold cross-validated model comparison provides independent, estimator-free evidence for this interpretation: the best unimodal MLP (mRNA) achieves AUC =0.928±0.013, comparable to or slightly above both a concatenation MLP (AUC =0.920±0.017) and the VMIB (AUC =0.915±0.016). The fact that multimodal fusion provides negligible gain over the strongest single modality corroborates the redundancy interpretation without relying on MI bound subtraction.

### 9.3. Cross-Level Modality Contrast: TCGA-GBMLGG

To examine whether the redundancy-dominated pattern generalizes or reflects the specific choice of same-level omics modalities, we repeat the information decomposition on a second TCGA cohort using cross-level modalities. The TCGA glioma dataset (GBMLGG, N=1042) pairs demographic features (age and sex; $d_{1}=2$) with molecular features (15 key gene mutations including IDH1, TP53, and ATRX, plus 80 copy-number variation features; $d_{2}=95$) for 6-class histological subtype classification (GBM, anaplastic astrocytoma, anaplastic oligoastrocytoma, astrocytoma, oligoastrocytoma, oligodendroglioma; H(Y)=1.76 nats). MI estimates are averaged over 5-fold stratified cross-validation (Table 4).

The synergy proxy S=−0.16±0.06 (from separate classifiers) is an order of magnitude smaller in absolute value than the BRCA omics pairs (S≈−0.63). Notably, a robustness check using a single VMIB model with modality dropout (which ensures consistent bound tightness across subsets) yielded S=+0.04, with the opposite sign. This sign sensitivity illustrates that *S* near zero is at the boundary of what the estimators can resolve, and the sign should not be over-interpreted. What *is* robust across estimation methods is that demographic and molecular features interact much more additively than same-level omics pairs: the absolute value |S| is consistently near zero for GBMLGG versus |S|≈0.6 for BRCA.

This cross-dataset comparison is consistent with the framework’s prediction that same-level modalities (e.g., multiple omics types measuring related transcriptional processes) tend toward redundancy, while cross-level modalities (e.g., phenotypic demographics and genotypic molecular markers) interact more additively. The distinction has practical implications for data acquisition: adding a redundant modality provides marginal gain through noise averaging, whereas adding a cross-level modality may yield unique predictive information.

### 9.4. Compression–Prediction Tradeoff in the Information Plane

**Figure 10:**
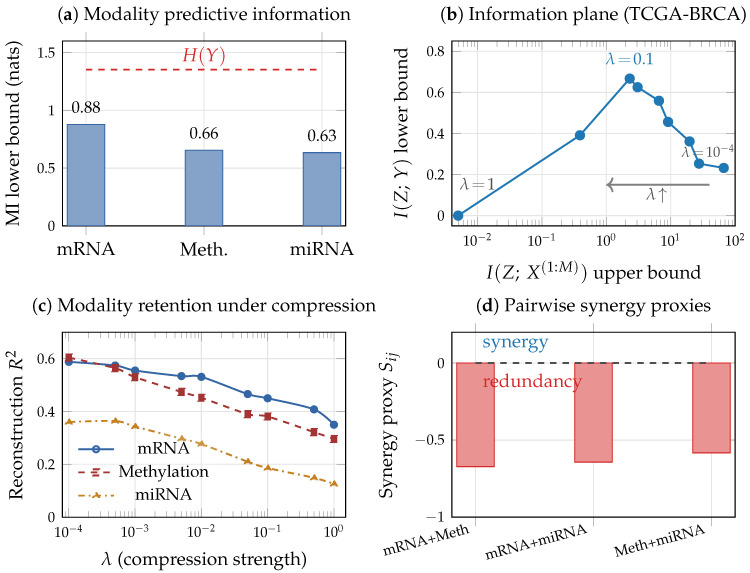
Information-theoretic analysis of TCGA-BRCA multi-omics data. (**a**) Predictive information per modality: mRNA provides the highest MI with the cancer subtype target (I≥0.878 nats, 64.9% of H(Y)); dashed line marks H(Y)=1.352 nats. (**b**) Empirical information plane trajectory as λ varies: the representation traces a compression–prediction tradeoff consistent with the theoretical predictions of Section 3, with optimal operating points near λ=0.05–0.1 (strong compression, near-maximal prediction). At λ=1, the representation collapses. (**c**) Per-modality retention (probing $R^{2}$ from *Z* to $X^{\left(i\right)}$): miRNA, the least predictive modality, is compressed most aggressively at all λ levels, consistent with the IB principle’s prediction that optimal representations preferentially preserve informative signal. (**d**) All pairwise interaction proxies are negative, consistent with redundancy-dominated interactions among omics modalities (see estimation caveats in Section 9.1).

Figure 10 shows the empirical information plane trajectory and per-modality retention as the compression parameter λ is varied across two orders of magnitude.

The trajectory in panel (b) exhibits the predicted behavior from Section 3: at weak compression ($\lambda =10^{-4}$), the representation retains substantial input information ($I(Z;X^{\left(1:M\right)})\leq 66.7$ nats) with suboptimal predictive efficiency; as λ increases to 0.05–0.1, the model finds a favorable operating point with strong compression ($I(Z;X^{\left(1:M\right)})\leq 2$–3 nats) while maximizing I(Z;Y)≥0.67 nats; at extreme compression (λ=1.0), the representation collapses. Panel (c) reveals that compression is applied non-uniformly: miRNA is compressed most aggressively ($R^{2}$ drops from 0.36 to 0.13), while mRNA retains the highest reconstruction fidelity across all λ values. This differential compression aligns with the information decomposition results and supports the IB principle’s prediction that optimal representations preferentially retain predictive signal while discarding less informative modalities. We note that the I(Z;Y) lower bound increases with moderate λ before declining at strong compression; this non-monotonicity reflects the regularization benefit of the KL penalty on generalization (lower test cross-entropy) rather than a violation of the theoretical compression–prediction tradeoff, and is consistent with known behavior of variational bounds in finite-sample regimes [23].

### 9.5. Missing-Modality Robustness

We train three model variants to evaluate the missing-modality framework of Section 5: (i) Standard: VMIB with λ=0.01, no robustness mechanisms; (ii) Modality dropout: VMIB with random modality zeroing (p=0.3) during training; (iii) Consistency: VMIB with the KL consistency penalty of Equation (15) (γ=0.05, warmup over 30 epochs). Table 5 reports the AUC under systematic modality ablation.

The results support the framework’s predictions. When mRNA (the dominant modality) is present, all models perform well regardless of which other modalities are missing ($I_{\mathrm{miss}}\approx 0$). The critical differences emerge when mRNA is absent: the standard model drops to AUC = 0.668 with miRNA alone and AUC = 0.725 with methylation + miRNA, while the dropout and consistency models degrade far more gracefully (AUC = 0.796 and AUC = 0.899, respectively). Notably, the modality dropout model achieves the highest full-data AUC (0.945) and the best robustness. Interestingly, simple modality dropout outperforms the explicit KL consistency penalty, suggesting that the stochastic training signal from randomly missing modalities provides an effective implicit regularizer that is easier to optimize than the explicit forward-KL objective of Equation (15). This finding motivates future work on tighter consistency objectives and adaptive penalty scheduling.

### 9.6. Fusion Collapse Diagnostics

**Figure 11:**
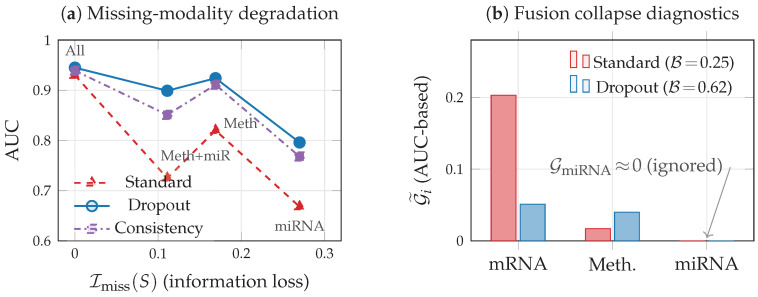
Missing-modality robustness and fusion collapse on TCGA-BRCA. (**a**) AUC as a function of theoretical information loss $I_{\mathrm{miss}}\left(S\right)$ for three model variants. Each point represents a modality subset (labeled); the standard model (red, dashed) degrades steeply when mRNA is absent, while the dropout (blue, solid) and consistency (purple, dash-dotted) models maintain substantially higher AUC. The vertical spread at similar $I_{\mathrm{miss}}$ values reflects that the information loss measure captures intrinsic data properties while model degradation depends on training strategy. (**b**) Empirical predictive gaps ${\tilde{G}}_{i}$ (AUC drop upon ablating modality *i*; averaged over three seeds), serving as proxies for the information-theoretic $G_{i}$ of Section 4.5. The standard model concentrates 92% of its predictive reliance on mRNA (${\tilde{G}}_{\mathrm{mRNA}}=0.203$; balance index B=0.25), effectively ignoring miRNA (${\tilde{G}}_{\mathrm{miRNA}}=0$). Modality dropout rebalances the representation: ${\tilde{G}}_{\mathrm{mRNA}}$ decreases to 0.051 while ${\tilde{G}}_{\mathrm{Meth}}$ increases to 0.040 (B=0.62), indicating more equitable utilization of available modalities.

Figure 11b shows the empirical modality-conditioned predictive gap ${\tilde{G}}_{i}=\mathrm{AUC}\left(\mathrm{all}\right)-\mathrm{AUC}\left(\mathrm{ablate}\;i\right)$, an operationally interpretable proxy for the information-theoretic quantity $G_{i}=I\left(Z;Y\right)-I\left(Z;Y|X^{\left(i\right)}\right)$ defined in Section 4.5. As noted there, both $G_{i}$ and ${\tilde{G}}_{i}$ are most informative in redundancy-dominated settings; in synergy-dominated tasks, they may underestimate the contribution of individually uninformative modalities. Under standard training, the model concentrates 92% of its total predictive reliance on mRNA (${\tilde{G}}_{\mathrm{mRNA}}=0.203$), with methylation contributing marginally (${\tilde{G}}_{\mathrm{Meth}}=0.017$) and miRNA contributing nothing (${\tilde{G}}_{\mathrm{miRNA}}=0$). The normalized balance index is B=0.25 (where 1.0 indicates perfect balance), confirming fusion collapse toward the dominant modality.

Increasing the IB compression parameter λ exacerbates fusion collapse: as λ increases from 0 to 0.1, the balance index decreases from 0.28 to 0.14 (Table 6). This is consistent with the theoretical prediction: stronger compression forces the encoder to retain only the most informative signal, which concentrates capacity on the dominant modality. In contrast, modality dropout at rate p=0.3 increases the balance index from 0.25 to 0.62 while simultaneously improving full-data AUC from 0.932 to 0.945. This shows that combating fusion collapse via the information consistency principle (Section 5) yields both more balanced representations and better predictive performance. We note that $G_{\mathrm{miRNA}}$ remains zero across all configurations, suggesting little to no detectable unique predictive contribution from miRNA beyond what mRNA and methylation already capture in this dataset. This is consistent with the low incremental MI $I(X^{\left(3\right)};Y|X^{\left(1\right)})\approx 0$ observed in Section 9.2 and illustrates that fusion collapse toward truly redundant modalities cannot be “corrected” by training alone, as the information-theoretic ceiling leaves no room for improvement.

### 9.7. Uncertainty Quantification and Selective Prediction

To illustrate the entropy-based uncertainty framework of Section 6, we evaluate three diagnostics on the TCGA-BRCA data: out-of-distribution (OOD) detection, calibration, and selective prediction via entropy-based deferral. For OOD detection, we train the VMIB on four of five BRCA subtypes and hold out the HER2-enriched class as a surrogate OOD population. The mean predictive entropy (Equation (16)) for in-distribution samples is ${\bar{H}}_{\mathrm{ID}}=0.133$, while OOD samples exhibit ${\bar{H}}_{\mathrm{OOD}}=0.271$, a 2.0× increase. This separation indicates that predictive entropy provides a viable OOD signal in multimodal biomedical settings, as argued in Section 6.2: distribution shift reduces I(Z;Y) under the test distribution, inflating predictive uncertainty. For calibration, we compute the expected calibration error (ECE). The multimodal VMIB achieves ECE=0.173, comparable to the unimodal mRNA baseline (ECE=0.166), suggesting that the fusion process does not introduce systematic miscalibration in this setting.

**Figure 12:**
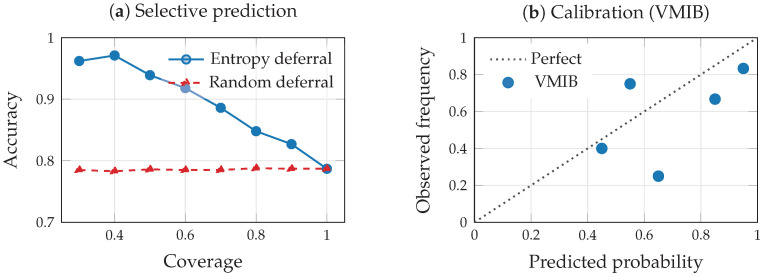
Uncertainty diagnostics on TCGA-BRCA. (**a**) Selective prediction via entropy-based deferral (blue, solid) substantially improves accuracy on retained predictions by routing uncertain cases to human review; random deferral (red, dashed) provides no improvement, confirming entropy as an informative uncertainty measure. (**b**) Reliability diagram for the VMIB model; the dotted diagonal represents perfect calibration. Computed from TCGA-BRCA test data.

For selective prediction, we implement the entropy-based deferral rule of Equation (18). At full coverage (100%), accuracy is 0.787. As the model defers the highest-entropy cases, accuracy on retained predictions improves monotonically: 0.886 at 70% coverage, 0.939 at 50%, and 0.962 at 30% (Table 7). Random deferral, by contrast, yields approximately constant accuracy (∼0.787) regardless of coverage, confirming that the improvement is attributable to the entropy ranking rather than selective subsampling. These results (Figure 12) demonstrate that the deferral rule produces clinically meaningful risk stratification: the model can reliably identify cases where its predictions are trustworthy and escalate uncertain cases to human review, consistent with the risk-sensitive decision framework of Section 6.4.

### 9.8. Longitudinal Disease Modeling: OASIS-2 Alzheimer’s Data

To illustrate the temporal information-theoretic concepts of Section 7 on longitudinal data, we use the OASIS-2 Alzheimer’s dataset [79], which contains 150 subjects scanned on two or more visits, for a total of 373 imaging sessions; in our preprocessing, this yields 223 consecutive visit pairs. Two modalities are available at each visit: a clinical measure (MMSE cognitive score) and a neuroimaging measure (nWBV, normalized whole-brain volume). The prediction target is the Clinical Dementia Rating (CDR) at the next visit, binarized as impaired (CDR>0) vs. normal (CDR=0); the label entropy is H(Y)=0.690 nats.

We first estimate transfer entropy (Equation (20)) to quantify directional modality influence on disease progression beyond the autoregressive baseline. Transfer entropy from MMSE to CDR is ${\mathrm{TE}}_{\mathrm{MMSE}\to \mathrm{CDR}}=+0.003$ nats, indicating a small positive contribution of cognitive scores beyond CDR history alone. The estimated transfer entropy from nWBV to CDR is −0.001 nats; since transfer entropy is a conditional MI and therefore nonnegative in theory, this value is consistent with zero within estimation noise, indicating negligible directional influence of brain volume on next-visit CDR beyond disease history. These modest values reflect the strong autoregressive structure of CDR: the current CDR alone predicts the next-visit CDR with 91.9% accuracy, leaving limited room for additional modality contributions. Notably, the per-stage analysis reveals that modality influence varies across the disease course. In early disease (CDR=0), nWBV has slightly higher discriminative power, while at the mild impairment stage (CDR=0.5), MMSE becomes more discriminative. This stage-dependent modality relevance is consistent with the theoretical prediction in Section 7 and the conceptual illustration in Figure 8b: different modalities carry maximal information at different points in disease progression.

**Figure 13:**
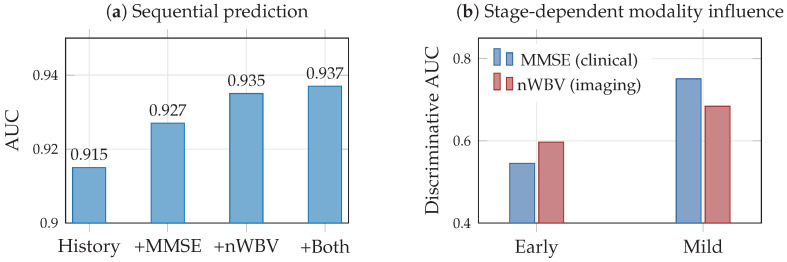
Longitudinal disease modeling on OASIS-2 Alzheimer’s data. (**a)** Sequential prediction AUC improves monotonically as modalities are added to the autoregressive baseline (CDR history only), with the multimodal model (both MMSE and nWBV) achieving AUC = 0.937. (**b**) Stage-dependent modality influence: in early disease (CDR = 0), brain volume (nWBV) has slightly higher discriminative power; in the mild stage (CDR = 0.5), the cognitive score (MMSE) becomes more discriminative, consistent with the temporal modality prioritization concept of Section 7. Computed from OASIS-2 data.

We further evaluate sequential prediction models to quantify the practical benefit of multimodal temporal modeling (Table 8). Model A, using CDR history only, achieves AUC = 0.915, establishing a strong autoregressive baseline. Adding MMSE (Model B) improves AUC to 0.927 (+0.012), while adding nWBV (Model C) yields AUC = 0.935 (+0.019). Combining both modalities (Model D) achieves AUC = 0.937 (+0.022). The improvement is small but consistent across subject-level 5-fold cross-validation, reflecting the high baseline predictability of dementia progression. These results (Figure 13) provide empirical grounding for the sequential information bottleneck framework of Section 7 (Equation (19)), demonstrating that multimodal temporal data provides measurable, if modest, predictive gains even when the autoregressive signal is dominant.

### 9.9. Pretraining and Adaptation Diagnostics

To illustrate the information-theoretic diagnostics proposed in Section 8 in a controlled setting, we evaluate pretraining dynamics, adaptation strategies, and robustness using a VMIB encoder pretrained on TCGA-BRCA for 200 epochs, then adapted to a downstream classification task with only 50 labeled samples. We note that this setup simulates the pretraining/adaptation workflow of foundation models at a smaller scale; evaluation of full-scale biomedical foundation models (e.g., BiomedCLIP, Med-PaLM) remains an important direction for future work.

The representation entropy $H_{\mathrm{enc}}$ of the encoder output tracks the training dynamics predicted by the multi-task IB framework. During early training, $H_{\mathrm{enc}}$ peaks at epoch 5 (∼490), reflecting an initial expansion phase in which the encoder captures broad input features. It then steadily decreases, converging to ∼271 by epoch 200, consistent with the compression phase shown in Figure 9a, where representations are progressively refined to retain task-relevant information. For adaptation, we compare four strategies against the pretrained baseline (AUC = 0.922). Training from scratch with 50 samples yields AUC = 0.911, underperforming the pretrained model and incurring high sensitivity shift (ΔKL=+2.18). Full fine-tuning achieves AUC = 0.936 with moderate sensitivity increase (ΔKL=+0.42). Linear probing, which freezes the encoder and trains only the classification head, achieves AUC = 0.928 with negligible sensitivity shift (ΔKL=−0.07), making it the most efficient strategy per unit of distributional perturbation. Partial fine-tuning (unfreezing only the final encoder layers) achieves the highest AUC of 0.964, though at the cost of greater sensitivity shift (ΔKL=+2.69). These results are consistent with the adaptation efficiency analysis in Figure 9c: linear probing offers the best efficiency (predictive gain per unit sensitivity increase), while partial fine-tuning trades efficiency for absolute performance (Table 9).

**Figure 14:**
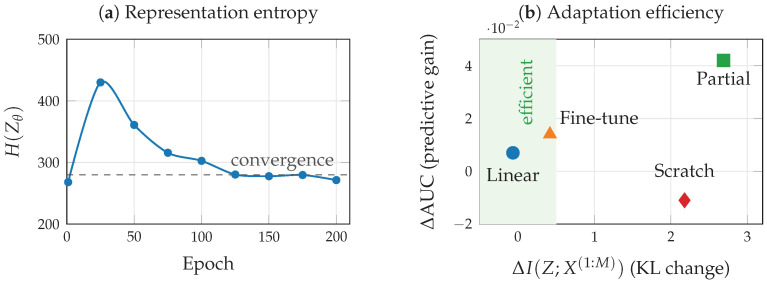
Foundation model diagnostics on TCGA-BRCA. (**a**) Representation entropy $H(Z_{\theta })$ during VMIB pretraining: entropy initially increases as the model learns richer representations, then converges to a plateau, consistent with the pattern predicted in Section 8.1. (**b**) Adaptation efficiency: linear probing achieves predictive gain with minimal sensitivity increase (efficient region, shaded), while training from scratch incurs large sensitivity increase with negative returns, consistent with Section 8.2. Computed from TCGA-BRCA data.

For missing-modality robustness, we compare a consistency-trained pretrained model against a standard pretrained model. The consistency-trained model exhibits 37% less average AUC degradation under missing modalities (0.049 vs. 0.078), validating the robustness diagnostic proposed in Figure 9d. Together, these experiments (Figure 14) demonstrate that the information-theoretic diagnostics of Section 8, including representation entropy tracking, adaptation efficiency via ΔKL, and consistency-based robustness, are computable on real biomedical data and provide actionable guidance for foundation model deployment.

### 9.10. Summary of Empirical Findings

The empirical case studies support predictions from each theoretical section of the framework across three biomedical datasets:

1. Information decomposition (Section 4): MI estimation identifies mRNA as the dominant modality in BRCA, reveals redundancy among same-level omics (S≈−0.63), and distinguishes cross-level near-additivity in GBMLGG (S≈−0.16). A 5-fold CV baseline comparison confirms that fusion provides negligible gain over the strongest single modality in redundancy-dominated settings.
2. Compression–prediction tradeoff (Section 3): The VMIB traces a clear trajectory in the information plane, with non-uniform per-modality compression that preferentially discards less informative modalities. The synthetic interaction model confirms genuine synergy detection ($S_{12}>0$ at α>0).
3. Missing-modality robustness (Section 5): Modality dropout reduces worst-case AUC degradation (from 0.668 to 0.796 for miRNA-only) while maintaining full-data performance.
4. Fusion collapse (Section 4): The empirical predictive gap ${\tilde{G}}_{i}$ detects that standard training concentrates 92% of reliance on a single modality; modality dropout increases the balance index from 0.25 to 0.62.
5. Uncertainty and selective prediction (Section 6): OOD samples exhibit 2.0× higher predictive entropy than ID samples. Entropy-based deferral raises accuracy from 0.787 to 0.939 at 50% coverage.
6. Longitudinal modeling (Section 7): Transfer entropy on OASIS-2 Alzheimer’s data reveals stage-dependent modality influence, and multimodal temporal models improve AUC by +0.022 over autoregressive baselines.
7. Foundation model diagnostics (Section 8): Representation entropy shows expected training dynamics. Linear probing achieves efficient adaptation (ΔAUC =+0.007 with minimal sensitivity increase), and consistency-trained models show 37% less missing-modality degradation.

These results show that the information-theoretic quantities developed in this paper are computable and interpretable across diverse biomedical settings. Their quantitative precision depends on the estimation method and sample size; stronger claims would require consistent MI estimators with formal guarantees, an important direction for future work.

## 10. Discussion and Open Problems

The information-theoretic framework developed in this paper unifies representation learning, robustness, uncertainty, and temporal modeling under a common entropy-based perspective. While it clarifies many empirical phenomena and provides principled design guidance, several fundamental challenges remain. We identify ten open problems, organized from foundational methodology through domain-specific extensions to clinical translation (Table 10).

From architecture-centric to principle-centric evaluation.

A central implication of this work is that many architectural choices can be interpreted as approximations to the same underlying MIB objective. An open problem is to develop architecture-independent evaluation protocols based on information-theoretic quantities [22,45]. While task accuracy remains important, it does not reveal whether performance arises from robust, generalizable information or from memorizing spurious correlations. Metrics such as H(Z), $I(Z;X^{\left(i\right)})$, and $G_{i}$ offer principled alternatives, but reliable estimation in high-dimensional settings remains challenging [23].

Causal information flow.

Mutual information and transfer entropy quantify statistical dependence and directional influence but do not establish causality. Integrating causal inference with the IB framework, for example by seeking representations that maximize I(Z;Y) subject to independence from known confounders Z⊥⊥C, is an important direction [18,54]. Extending transfer entropy to settings with latent confounding and irregular sampling, common in longitudinal biomedical data, poses additional challenges.

High-dimensional information estimation.

The quantities central to this framework are notoriously difficult to estimate in high-dimensional, continuous spaces [23,45]. Variational bounds provide practical surrogates, but their tightness depends on model expressivity and optimization quality. Characterizing the bias–variance trade-offs of variational information estimators in biomedical regimes with limited samples and heterogeneous modalities is essential for principled application.

Privacy–utility trade-offs.

Information theory naturally formalizes privacy constraints through bounds of the form $I(Z;X_{\mathrm{private}}^{\left(i\right)})\leq \delta$, limiting leakage of protected attributes while maintaining predictive performance [80]. Open problems include jointly optimizing for sufficiency, compression, and privacy, and understanding how different modalities leak sensitive information at different rates.

Multi-scale biological complexity.

Biological systems operate across molecular, cellular, organ, and population scales. An open challenge is to develop hierarchical information bottlenecks that learn multi-level representations $\left\{Z^{\left(1\right)},Z^{\left(2\right)},\ldots \right\}$ satisfying $I\left(Z^{\left(k\right)};Y\right)\geq I\left(Z^{\left(k+1\right)};Y\right)$ and $I\left(Z^{\left(k\right)};X\right)\geq I\left(Z^{\left(k+1\right)};X\right)$, explicitly modeling information flow across scales and enabling principled integration of multi-omics with imaging and clinical records [2,3].

Fairness and algorithmic equity.

Biomedical AI systems must provide equitable performance across demographic groups. Information theory offers natural tools for formulating fairness constraints: requiring I(Z;A)≤δ for a protected attribute *A* (e.g., race, sex) enforces demographic parity in the representation space, while preserving I(Z;Y) ensures that predictive performance is maintained [81,82]. The tension between fairness and utility mirrors the compression–prediction trade-off in the IB framework, suggesting that information-theoretic Pareto analysis can guide the design of equitable multimodal systems. Open questions include how fairness constraints interact across modalities (for instance, whether clinical text and imaging encode demographic information at different rates) and how to balance group-level and individual-level fairness desiderata within the IB formalism.

Federated and distributed multimodal learning.

In practice, multimodal biomedical data are often distributed across institutions that cannot share raw data due to privacy regulations. Federated learning enables collaborative model training without centralizing data [83,84], but multimodal federation poses unique challenges: different sites may collect different subsets of modalities, and data heterogeneity across institutions introduces distribution shifts that compound the missing-modality problem. From an information-theoretic perspective, federated multimodal learning requires controlling information leakage $I(Z;X_{\mathrm{private}}^{\left(i\right)})$ across institutional boundaries while preserving sufficient predictive information I(Z;Y). Understanding how to decompose and aggregate partial information across sites, without compromising either privacy or prediction, remains an open problem.

Domain adaptation and distribution shift.

Biomedical AI systems trained at one institution often degrade when deployed at another due to distribution shifts in imaging protocols, patient demographics, laboratory assays, and clinical workflows [85,86]. Information theory provides a natural framework for quantifying and addressing these shifts: the KL divergence $\mathrm{KL}(p_{\mathrm{target}}∥p_{\mathrm{source}})$ measures the information-theoretic cost of distribution mismatch, while domain-invariant representations can be formalized through constraints of the form I(Z;D)≤ϵ, where *D* indexes the domain. Extending these ideas to multimodal settings, where different modalities may shift at different rates and in different directions, is an important open problem that connects the IB framework to the domain adaptation literature.

Active and adaptive data acquisition.

The information-theoretic framework naturally supports principled decisions about which modalities to acquire for a given patient. The conditional mutual information $I(X^{\left(i\right)};Y|X^{\left(S\right)})$ quantifies the expected predictive value of modality *i* given already-observed data $X^{\left(S\right)}$, providing a cost-aware acquisition criterion: acquire the modality that maximizes information gain per unit cost [18,63]. In longitudinal settings, the transfer entropy profiles of Section 7 suggest that different modalities should be prioritized at different disease stages, enabling adaptive monitoring protocols. Open problems include optimizing sequential acquisition under budget constraints, handling the combinatorial explosion of modality subsets, and incorporating patient-specific risk profiles into acquisition policies.

Regulatory and clinical translation.

Measures such as predictive entropy and conditional information loss provide transparent indicators of model reliability that could complement existing validation standards. However, translating these into actionable guidelines for clinicians and regulators, including standardized thresholds, reporting practices, and monitoring tools for deployed systems, remains an open problem [13,14].

## 11. Conclusions

This paper has presented a unified information-theoretic foundation for multimodal biomedical machine learning, framing learning, fusion, robustness, uncertainty, and temporal modeling as manifestations of a single underlying principle: the control and optimization of information. At the core of the framework is the multimodal information bottleneck objective

$$\min\;I\left(Z;X^{\left(1:M\right)}\right)-\beta \;I\left(Z;Y\right),$$

which provides a unifying lens for analyzing representation learning across heterogeneous biomedical modalities. The variational VMIB formulation makes this objective tractable and connects it to practical learning algorithms, while extensions to missing-modality consistency and sequential learning reveal how robustness, generalization, and graceful degradation arise naturally from information-theoretic constraints rather than ad hoc heuristics.

By decomposing predictive information across modalities, we showed how redundancy, synergy, and modality domination can be quantified through operationally grounded proxies and the fusion collapse diagnostic $G_{i}$. The treatment of uncertainty and calibration in terms of entropy and the cross-entropy gap connects predictive confidence to fundamental limits on information availability, while the extension to longitudinal settings via transfer entropy and the sequential IB enables principled modeling of disease progression and temporal modality prioritization. Applications to multimodal foundation models provide architecture-agnostic diagnostics for representation quality, modality balance, and information-efficient adaptation.

Empirical case studies across three biomedical datasets showed that the framework’s key quantities are computable and interpretable on real data. On TCGA cancer data, MI decomposition identified modality dominance and redundancy (S≈−0.63 for same-level omics, S≈−0.16 for cross-level modalities), the VMIB traced a compression–prediction tradeoff in the information plane, and the empirical predictive gap ${\tilde{G}}_{i}$ detected modality dominance correctable through modality dropout. Entropy-based selective prediction raised accuracy from 0.787 to 0.939 at 50% coverage. On OASIS-2 Alzheimer’s data, transfer entropy revealed stage-dependent modality influence and multimodal temporal models improved prediction by +0.022 AUC. Pretraining/adaptation diagnostics showed that lightweight adaptation achieves efficient predictive gains while consistency training reduces missing-modality degradation by 37%. We note that these experiments illustrate the framework’s applicability; the precision of individual MI estimates depends on the estimation method and sample size, and stronger claims would require purpose-built consistent estimators with formal guarantees.

Looking forward, we have identified ten open research frontiers, spanning high-dimensional information estimation, causal information flow, fairness, federated learning, domain adaptation, and active data acquisition, where information-theoretic tools can guide principled solutions to pressing challenges in biomedical AI. Ultimately, multimodal biomedical machine learning is not merely a problem of fusing data sources, but one of principled information control: determining what to preserve, what to discard, and how uncertainty propagates under incomplete and evolving data. By grounding these questions in information theory, this paper aims to provide a useful theoretical foundation for the next generation of multimodal biomedical AI systems.

## Figures and Tables

**Table 1:** Summary of notation and key information-theoretic quantities.

Symbol	Description
$X^{\left(i\right)}$	Modality *i* random variable, i=1,…,M
*Y*	Prediction target (diagnosis, prognosis, risk)
*Z*	Latent representation
H(X)	Shannon entropy of *X*
H(Y∣X)	Conditional entropy of *Y* given *X*
I(X;Y)	Mutual information between *X* and *Y*
$I(X^{\left(i\right)};Y|X^{\left(j\right)})$	Conditional MI: incremental value of modality *i* given *j*
KL(p∥q)	Kullback–Leibler divergence from *q* to *p*
${\mathrm{TE}}_{X\to Y}$	Transfer entropy from process *X* to process *Y*
$D_{f}\left(p∥q\right)$	*f*-divergence from *q* to *p*
$H_{\alpha }\left(X\right)$	Rényi entropy of order α
β,λ,γ	Trade-off parameters (IB, compression, consistency)

**Table 2:** Mapping existing multimodal architectures to the MIB/VMIB framework. Each architecture implicitly controls compression and prediction through different mechanisms.

Architecture	Implicit $I(Z;X^{\left(1:M\right)})$ Control	Implicit I(Z;Y) Target	Framework Interpretation
Early fusion + bottleneck	Dimension reduction via bottleneck layer	Supervised cross-entropy or regression loss	Direct MIB approximation
Late fusion (ensemble)	Independent per-modality compression	Per-modality supervised losses	Factored MIB with separate encoders
Cross-attention transformer	Attention sparsity and head pruning	Supervised loss on fused representation	Soft modality selection; dynamic info. allocation
Contrastive (e.g., CLIP)	Low-dim. projection	Cross-modal alignment objective	Compression via projection; prediction via alignment
Graph-based (e.g., MOGONET)	Graph pooling and neighborhood aggregation	Node/graph classification loss	Topological compression; structure-aware MIB

**Table 3:** Information decomposition on TCGA-BRCA (5-fold stratified cross-validation; mean ± std across folds). MI lower bounds $I(X^{\left(S\right)};Y)$ for individual modalities and combinations, with classification accuracy and AUC. All pairwise interaction proxies $S_{ij}$ are negative, consistent with redundancy-dominated modality interactions (see estimation caveats in Section 9.1).

Modality Subset $X^{\left(S\right)}$	MI Bound (Nats)	% of H(Y)	Acc.	AUC
mRNA	0.878±0.044	64.9%	0.829	0.954
DNA methylation	0.655±0.098	48.4%	0.730	0.912
miRNA	0.634±0.011	46.9%	0.737	0.909
mRNA + methylation	0.861±0.056	63.6%	0.811	0.950
mRNA + miRNA	0.870±0.032	64.3%	0.815	0.954
Methylation + miRNA	0.707±0.071	52.2%	0.749	0.923
All three modalities	0.842±0.036	62.2%	0.806	0.949
Synergy proxies $S_{ij}=I\left(X^{\left(i\right)},X^{\left(j\right)};Y\right)-I\left(X^{\left(i\right)};Y\right)-I\left(X^{\left(j\right)};Y\right)$				
$S_{\mathrm{mRNA},\mathrm{meth}}$	−0.67	redundancy-dominated		
$S_{\mathrm{mRNA},\mathrm{miRNA}}$	−0.64	redundancy-dominated		
$S_{\mathrm{meth},\mathrm{miRNA}}$	−0.58	redundancy-dominated		

**Table 4:** Information decomposition on TCGA-GBMLGG (5-fold stratified cross-validation; mean ± std across folds). Cross-level modalities (demographic and molecular) show near-additive interaction (S≈−0.16), in sharp contrast to the strong redundancy (S≈−0.63) among same-level omics modalities in TCGA-BRCA (Table 3).

Modality	MI Bound (Nats)	% of H(Y)	Acc.
Demographic (age, sex)	0.160±0.024	9.0%	0.359
Molecular (mutations + CNV)	0.719±0.038	40.7%	0.634
Demographic + Molecular	0.722±0.078	40.9%	0.641
Synergy proxy *S*	−0.16±0.06	weakly redundant, nearly additive	

**Table 5:** Missing-modality robustness on TCGA-BRCA. AUC (macro-averaged, one-vs-rest) under systematic modality ablation. $I_{\mathrm{miss}}\left(S\right)$ is the estimated information loss from missing modalities. Both robustness strategies (modality dropout and consistency penalty) substantially reduce degradation compared to the standard model, particularly for the most challenging scenarios (single remaining modality).

Available Modalities	$I_{\mathrm{miss}}$	Standard	Dropout	Consistency	ΔAUC
All three (full)	0.000	0.930	**0.945**	0.939	N/A
mRNA + methylation	0.000	0.963	0.963	**0.964**	<0.02
mRNA + miRNA	0.015	**0.913**	0.902	0.894	<0.05
Methylation + miRNA	0.111	0.725	**0.899**	0.851	0.17
mRNA only	0.000	**0.965**	0.955	0.958	<0.01
Methylation only	0.169	0.820	**0.924**	0.911	0.10
miRNA only	0.270	0.668	**0.796**	0.768	0.13

Bold: best AUC across the three methods for each row. ΔAUC: maximum AUC gap between Standard and best robust model under partial observation.

**Table 6:** Fusion collapse progression under IB compression and mitigation via modality dropout. $G_{i}$ values are AUC-based predictive gaps averaged over three random seeds. The balance index B is the normalized entropy of the $G_{i}$ distribution (1.0 = perfectly balanced). Stronger compression exacerbates mRNA dominance; modality dropout restores balance while improving AUC.

	Setting	$G_{\mathrm{mRNA}}$	$G_{\mathrm{Meth}}$	$G_{\mathrm{miRNA}}$	B	AUC
Compression	λ=0 (no IB)	0.179	0.019	0.000	0.28	0.950
	λ=0.001	0.202	0.028	0.000	0.34	0.920
	λ=0.01	0.203	0.017	0.000	0.25	0.932
	λ=0.1	0.194	0.007	0.000	0.14	0.926
Dropout	$p_{\mathrm{drop}}=0.1$	0.099	0.039	0.000	0.54	0.935
	$p_{\mathrm{drop}}=0.2$	0.074	0.041	0.000	0.59	0.939
	$p_{\mathrm{drop}}=0.3$	0.051	0.040	0.000	0.62	0.945

**Table 7:** Uncertainty-based diagnostics on TCGA-BRCA. OOD detection holds out one class (HER2-enriched) during training; selective prediction defers highest-entropy cases to improve accuracy on retained predictions.

Diagnostic	Metric	Value
*OOD detection (held-out class)*		
In-distribution entropy	$\bar{H}$	0.133
Out-of-distribution entropy	$\bar{H}$	0.271
Entropy ratio (OOD/ID)		2.0×
*Calibration (ECE)*		
VMIB (multimodal)	ECE	0.173
MLP (mRNA only)	ECE	0.166
*Selective prediction (entropy-based deferral)*		
Coverage 100%	Accuracy	0.787
Coverage 70%	Accuracy	0.886
Coverage 50%	Accuracy	0.939
Coverage 30%	Accuracy	0.962

**Table 8:** Longitudinal disease modeling on OASIS-2 Alzheimer’s data. Transfer entropy (TE) measures directional modality influence on disease progression beyond CDR history. Sequential prediction models add modalities to the autoregressive baseline (subject-level 5-fold CV, logistic regression).

Model/Measure	AUC	ΔAUC
*Transfer entropy (nats)*		
${\mathrm{TE}}_{\mathrm{MMSE}\to \mathrm{CDR}}$	+0.003	
${\mathrm{TE}}_{\mathrm{nWBV}\to \mathrm{CDR}}$	≈0 (−0.001 *)	
*Sequential prediction*		
A: CDR history only	0.915	(baseline)
B: +MMSE (clinical)	0.927	+0.012
C: +nWBV (imaging)	0.935	+0.019
D: +both modalities	0.937	+0.022

* Negative value is estimation noise; TE is nonneg. in theory.

**Table 9:** Foundation model diagnostics on TCGA-BRCA. Adaptation strategies are evaluated using 50 labeled samples from a held-out split. ΔAUC and ΔKL are relative to the pretrained baseline. Missing-modality robustness compares consistency-trained vs standard pretrained models.

Strategy	AUC	ΔAUC	ΔKL
*Adaptation efficiency (50 labeled samples)*			
Pretrained baseline	0.922	N/A	N/A
From scratch	0.911	−0.011	+2.18
Linear probe	0.928	+0.007	−0.07
Full fine-tune	0.936	+0.014	+0.42
Partial fine-tune	0.964	+0.042	+2.69
*Missing-modality robustness (avg AUC degradation)*			
MFM + consistency	0.049		
MFM standard	0.078		

**Table 10:** Open problems and their information-theoretic hooks within the unified framework.

Open Problem	Key Information-Theoretic Hook
Architecture-independent evaluation	H(Z), $I(Z;X^{\left(i\right)})$, $G_{i}$ as standardized metrics
Causal information flow	Causal IB: max I(Z;Y) s.t. Z⊥⊥C
High-dimensional estimation	Tightness of variational MI bounds
Privacy–utility trade-offs	$I(Z;X_{\mathrm{priv}}^{\left(i\right)})\leq \delta$ leakage constraints
Multi-scale hierarchical IB	Nested $Z^{\left(k\right)}$ with monotone $I(Z^{\left(k\right)};Y)$ across scales
Fairness and equity	I(Z;A)≤δ; IB Pareto fairness–utility analysis
Federated learning	Distributed information decomposition under privacy
Domain adaptation	$\mathrm{KL}(p_{t}∥p_{s})$ shift; I(Z;D)≤ϵ invariance
Active data acquisition	$I(X^{\left(i\right)};Y|X^{\left(S\right)})$ as cost-aware acquisition criterion
Regulatory translation	Predictive entropy and $I_{\mathrm{miss}}$ as transparency metrics

## Data Availability

The empirical studies in Section 9 use three publicly available datasets: (1) the TCGA-BRCA multi-omics dataset as preprocessed by Wang et al. [8] (available at https://github.com/txWang/MOGONET (accessed on 15 February 2026)); (2) the TCGA-GBMLGG clinical and genomic dataset from Chen et al. [87] (available at https://github.com/mahmoodlab/MCAT (accessed on 15 February 2026)); and (3) the OASIS-2 longitudinal Alzheimer’s MRI dataset [79] (available at https://www.kaggle.com/datasets/jboysen/mri-and-alzheimers (accessed on 15 February 2026)). No new primary data were generated.

## Abbreviations

The following abbreviations are used in this manuscript:

AI	Artificial intelligence
AUC	Area under the receiver operating characteristic curve
BRCA	Breast cancer (TCGA cohort designation)
CLIP	Contrastive language–image pre-training
DPI	Data processing inequality
CDR	Clinical Dementia Rating
CNV	Copy number variation
ECE	Expected calibration error
EHR	Electronic health record
GBMLGG	Glioblastoma and lower-grade glioma (TCGA cohort designation)
IB	Information bottleneck
KL	Kullback–Leibler
MFM	Multimodal foundation model
LoRA	Low-rank adaptation
MI	Mutual information
MLP	Multilayer perceptron
MMSE	Mini-Mental State Examination
MIB	Multimodal information bottleneck
MINE	Mutual information neural estimation
ML	Machine learning
OASIS	Open Access Series of Imaging Studies
OOD	Out-of-distribution
PAM50	Prediction Analysis of Microarray 50 (breast cancer subtype classifier)
PID	Partial information decomposition
SIB	Sequential information bottleneck
TCGA	The Cancer Genome Atlas
TE	Transfer entropy
VMIB	Variational multimodal information bottleneck
